# Effectiveness of exercise intervention in improving physical and mental status of patients with alcohol use disorders: A systematic review and meta-analysis

**DOI:** 10.1371/journal.pone.0311166

**Published:** 2024-10-30

**Authors:** Jihai Li, Zhidong Zhou, Gang Gao, Liuhong Zang

**Affiliations:** 1 Institute of Physical Education, Xinjiang Normal University, Urumqi, Xinjiang, China; 2 Institute of Physical Education, Jishou University, Jishou, Hunan, China; Hamasaki Clinic, JAPAN

## Abstract

**Objectives:**

This meta-analysis and systematic review examined the effects of an exercise intervention on alcohol dependence and physical and mental states in patients with alcohol use disorder (AUD).

**Data sources:**

PubMed, Web of Science, Cochrane Library, EBSCO, and Embase.

**Study inclusion and exclusion criteria:**

Randomized controlled trials published in English from the inception of the database until June 30, 2024, were included. All forms of exercise intervention (aerobic, resistance, yoga, mixed exercise, etc.) were included in the study, using an exercise intervention for patients with AUD and a non-exercise control group. Studies that excluded acute exercise or did not describe a specific intervention program; duplicate publications; review articles, conference articles, etc.; and studies that did not report appropriate outcome metrics.

**Data extraction:**

This protocol was prepared according to the Preferred Reporting Items for Systematic Reviews and Meta-Analyses protocol standard. The risk of bias was assessed via the Cochrane risk-of-bias tool as described by the Cochrane Handbook for Systematic Reviews and Interventions.

**Data synthesis:**

Alcohol dependence (number of drinks per day, number of drinks per week, AUDIT), physical and mental status [maximal oxygen uptake (VO^2^ max), resting heart rate, anxiety state, depression state, stress level].

**Results:**

Seventeen RCTs with a total of 1,905 patients with alcohol use disorders were included as subjects, and the results revealed that the exercise intervention had a significant effect on alcohol dependence, the number of drinks per day and the AUDIT score, the exercise intervention also had a significant effect on physical and mental status, VO^2^max, the resting heart rate, the anxiety state, the depression state, and the stress level. High heterogeneity in the number of drinks per day, anxiety, depression and stress outcomes (I^2^ > 50%), but Egger’s test showed no publication bias for all outcome indicators (p > 0.05).

**Conclusions:**

Exercise intervention can effectively reduce alcohol dependence and significantly improve the physical and mental states of AUD patients, and exercise intervention as an adjunct to the treatment of AUD patients is significantly effective.

## Introduction

Alcohol use disorder (AUD) is one of the most prevalent and damaging mental illnesses [1]. It is estimated that more than 100 million people around the world suffer from AUD, and alcohol abuse is one of the leading causes of disability and death, with 23 million deaths from AUD worldwide in 2016 alone, accounting for 5.3% of all deaths [2–5]. The pathology of AUD is characterized by uncontrolled alcohol consumption, and the incidence of cardiovascular disease, diabetes mellitus, and various metabolic disorders is generally higher in AUD patients than in the normal population, due to the prolonged and heavy consumption of alcohol [6–8]. At the same time, AUD patients have a generally higher prevalence of other psychiatric disorders, especially anxiety, depression, and stress disorders, which often coexist with AUD [9,10]. Alcohol use disorders also have a social impact, with $249 billion in damages caused by excessive drinking in the United States in 2010, including lost productivity in all workplaces (72%), medical costs (11%), and property damage such as traffic accidents [11].

Despite the high prevalence of AUD and the large number of patients, the rate of AUD patients seeking medical care is very low, only about 20%, for a variety of reasons [12,13]. Moreover, not only the low percentage of AUD patients seeking medical treatment, but also the relapse rate of AUD patients is very high, and the first-year relapse rate is as high as 90% among AUD patients seeking treatment. Given the dual effects of a low rate of medical treatment and a high rate of relapse, the burden of alcohol use disorders on society is increasing, and there is an urgent need to seek alternative means to assist in the clinical treatment of AUD, improvement of physical and psychological status of AUD patients and reduction of recurrence of AUD symptoms. Physical activity has been shown to affect AUD both psychologically and physiologically; physiologically, exercise can increase beta-endorphin levels, which are associated with post-exercise euphoria, and exercise may also mitigate the negative effects of ethanol intake through cellular-level mechanisms, such as slowing the decline of hepatic mitochondria and mitigating oxidative damage [14,15]. At the same time, physical activity can improve mental performance and increase self-confidence, as well as bring about a relaxing and calming effect, helping to reduce psychological dependence on alcohol [12,16–21]. We also analyzed the published meta-analyses of exercise intervention for AUD [22–24], and the studies were not consistent in determining the effect of exercise intervention for AUD, while some of the studies were published earlier and included less literature, which did not form a strong enough evidence effect. Therefore, it is necessary to include RCTs from recent years for a new meta-analysis to provide evidence for the improvement effect of exercise intervention for AUD.

Although many experiments have verified the effects of physical activity interventions on alcohol dependence and improving the physical and mental states of AUD patients [25–31], the conclusions are still controversial. Therefore, the present study was conducted to collect the results of RCTs on exercise interventions for AUD patients and to provide strong evidence of alcohol dependence and physical and mental health in AUD patients through meta-analysis.

## Methods

This review was conducted in accordance with the Preferred Reporting Items for Systematic Reviews and Meta-Analyses (PRISMA), see S1 Checklist [32]. The review protocol was registered with the International Prospective Register of Systematic Review (PROSPERO) (CRD42024566795).

### Inclusion and exclusion criteria

The inclusion criteria were as follows: (1) the study was a randomized controlled trial, and the language restriction was English; (2) the intervention group included patients with DSM-5 diagnosed alcohol use disorder (AUD), patients with alcohol use disorder (AUD) treated in hospitals, patients with post-traumatic stress disorder (PTSD) with symptoms of AUD; (3) the experimental group and the control group were the same as the patients with alcohol use disorder (AUD); The control group was not scheduled for exercise and continued to receive regular treatment or maintain a normal life. The experimental group underwent planned exercise interventions of various types, intensities and durations for a minimum of two weeks while maintaining their regular treatment or life; (4) Outcome measurements: number of drinks per day, number of drinks per week, AUDI, VO^2^max, resting heart rate, anxiety state, depression state, and stress level. The exclusion criteria were as follows: (1) studies that did not describe a specific intervention program or acute exercise; (2) low-quality studies and duplicate publications; (3) conference reports, dissertations, review articles, and meta-analyses; and (4) studies that did not report the appropriate outcome measurements.

### Search strategy

A computerized search of the PubMed, Web of Science, Cochrane Library, EBSCO, and Embase databases was conducted by two researchers to collect RCTs of exercise interventions for alcohol dependence and physical and psychological status in patients with alcohol use disorders from the time the database was created until June 30, 2024. For searches in PubMed/Cochrane and Embase, terms from MeSH and Emtree, respectively, were used. In addition, references to the included literature were traced to supplement the acquisition of relevant literature. The search terms included: "alcoholism" or "alcohol related" or "alcohol dependence" or "alcohol use disorder" or "hazardous drinking" or "harmful drinking" or "alcohol abuse" or "alcohol addiction" or "binge drinking" or "heavy drinking" and "exercise" or "training" or "physical activity" or "aerobic activity" or "resistance training" or "aerobic combined with resistance training" or "sport" and "randomized controlled trial "randomized" or "randomized" or "placebo" or "RCT" (S1 Table in S1 Appendix).

### Study selection and data extraction

Two researchers independently screened the literature, extracted the characteristics of the literature, and then cross-checked the results. In cases of disagreement, a third researcher was consulted to assist in the judgment, and the corresponding authors were contacted as much as possible for the literature that lacked content. When screening the literature, we first excluded irrelevant literature by reading the titles and abstracts, and then read the remaining literature to understand the characteristics of the literature to determine whether it should be included in the final analysis. The extracted data included the first author, country, year of publication, target population (sample size, sex and age characteristics of each group), intervention measures (exercise mode, intensity, duration, period and frequency of a single intervention) and outcome measurements. All data extracted for this study are provided in the (S1 Dataset).

### Risk of bias

The risk of bias of the included studies was evaluated by 2 investigators according to the Cochrane Handbook Risk of Bias Assessment Tool (ROB 2)for RCTs [33], and in cases of disagreement, a third investigator was sought to assist in the judgment.

The items evaluated included randomization process, deviations from intended interventions, missing outcome data, measurement of the outcome, and selection of the reported result. Quality assessment charts were created via Review Manage (version 5.3) software. Also, we analyzed the risk of bias for each of the primary outcomes based on ROB 2.

### Certainty of evidence

Two researchers assessed the strength of evidence based on the Grading of Recommendation, Assessment, Development and Evaluation (GRADE) [34]. The strength of evidence was high at the beginning of the evaluation because all included studies were randomized controlled studies. Evidence levels were downgraded based on the results of Risk of bias, Inconsistency, Indirectness, Imprecision, and Publication bias. If the majority of the evidence (>50%) was from studies with a low risk of bias, the risk of bias (as assessed by RoB 2) limitation was considered not serious and was not downgraded. If the heterogeneity of the results was large (I^2^ >50%), it was downgraded one level. The results of this study are all from direct comparisons and none of this article is downgraded. Downgraded one level if the sample size of the outcome indicator was <400. Downgraded one level if there was publication bias in the Egger’s test (p < 0.05) for the outcome.

### Statistical analyses

The outcome indicators included in this paper are all continuous variables, because some indicators have different measurement units and methods, Standardized mean difference (SMD) was chosen as the effect indicator, and the random effect model was used to realize the combination of effect sizes and its 95% confidence interval (CI) calculations in Stata (version 17.0) software. The effect sizes were determined to be small when the SDM = 0.2 to 0.5, medium when the SMD = 0.5 to 0.8, and large when the SMD > 0.8. The heterogeneity of the outcomes was examined by the p-value and I^2^ value; if p was >0.1 and I^2^ <50%, the heterogeneity of the included studies was low; otherwise, the heterogeneity was considered high. Egger’s test was used to evaluate the publication bias of the literature for the outcome indicators, and if p < 0.05, the experiment was considered to have publication bias. In the present study, each outcome indicator was analyzed according to the type of exercise (aerobic, resistance, yoga, and mixed), exercise intensity, which was assessed in reference to existing studies [35] (high, medium, and low), the length of a single intervention (30 min or less, 30–60 min, and >60 min), the duration of the intervention period (12 weeks or less, and >12 weeks), and the form of exercise (groups, individuals), were analyzed separately. Statistically significant differences were defined as those for which P<0.05.

## Results

### Search results

**Fig 1:**
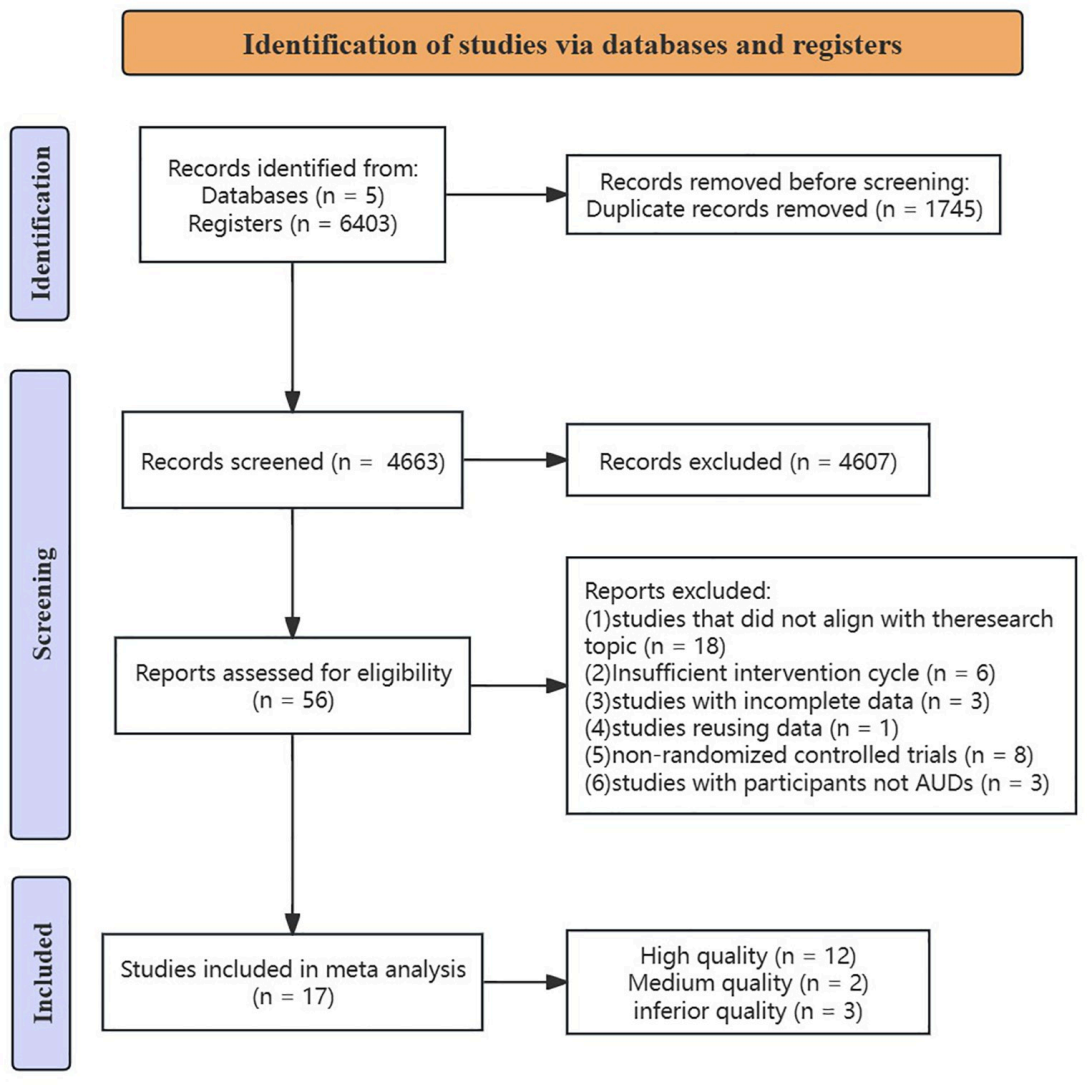
PRISM flow diagram.

A database search yielded 6403 relevant publications, including: PubMed (*n* = 331), Web of Science (*n* = 3359), the Cochrane Library (*n* = 1728), Embase (*n* = 589) and EBSCO (*n* = 396); 5 documents were obtained through other means, for a total of 6408 documents. A total of 1745 duplicates were excluded, 4607 were excluded by reading the title and abstract, and 39 were excluded by obtaining the full text to read (S3 Table in S1 Appendix), of which 18 were incompatible with the study design, 3 had an intervention period of less than 2 weeks, 3 could not extract the data, 1 was duplicated in a publication, 8 were non-randomized controlled trials, 3 had non-alcohol-use-disordered patients as the experimenters, and 17 RCTs were ultimately included (Fig 1) [25–30,36–46].

### Characteristics of the included trials

The basic characteristics of the included studies are shown (S2 Table in S1 Appendix). All of the included trials were randomized controlled trials, with 8 trials from the United States, 4 from Asia (2 from India and 2 from Turkey), and 5 from Europe (1 from Greece, 2 from Sweden, and 2 from Denmark). The year of publication for 82% of the included studies was within the last 10 years and 53% were within the last 5 years. Among these, 4 articles reported the number of drinks per day (with a sample size of 189 in the exercise intervention group and 109 in the non-exercise group), the number of drinks per week reported by 4 articles (153 in the exercise intervention group and 106 in the non-exercise group), AUDIT score reported by 4 articles (139 in the exercise intervention group and 991 in the non-exercise group), the VO^2^max score reported by 4 articles (110 in the exercise intervention group and 93 in the non-exercise group), the resting heart rate reported by 2 articles (41 in the exercise intervention group and 27 in the non-exercise group), the anxiety score reported by 6 articles (200 in the exercise intervention group and 150 in the non-exercise group), the depression score reported by 7 articles (254 in the exercise intervention group and 204 in the non-exercise group) and the stress score reported by 2 articles (65 in the exercise intervention group and 65 in the non-exercise group).

### Exercise interventions

The interventions included in the experiment included aerobic exercise (slow walking, lifestyle physical activity, jogging, swimming, etc.), resistance exercise, yoga, and combined exercise (aerobic and resistance [46], yoga and Pilates, and aerobic and basketball [45]); the intensity of the exercise was divided into three levels of intensity, high, medium and low; and the duration of a single intervention ranged from 15 min-75 min, with the main focus on the range of 30–60 min. The intervention period ranged from 3 weeks to 24 weeks, with the main focus on 12 weeks.

### Risk of bias

**Fig 2:**
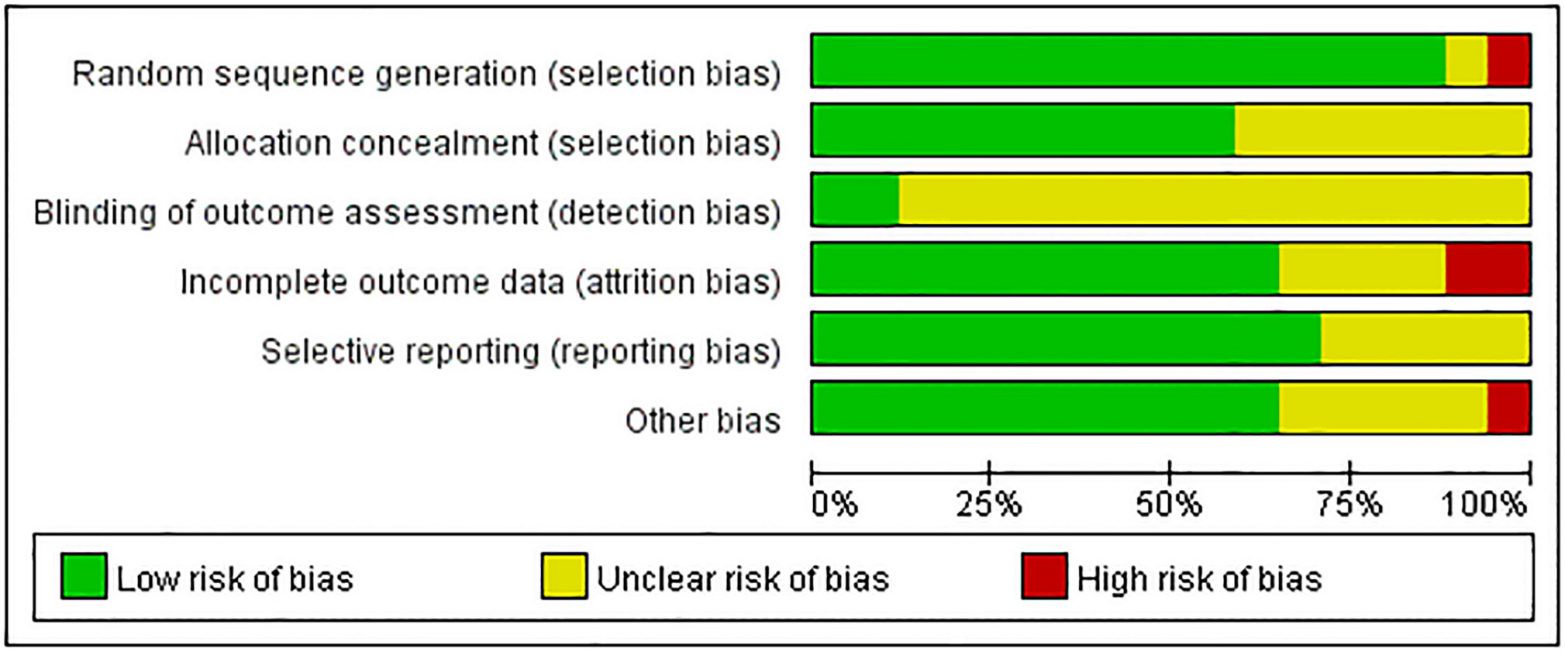
Bias assessment chart of the included literature.

**Fig 3:**
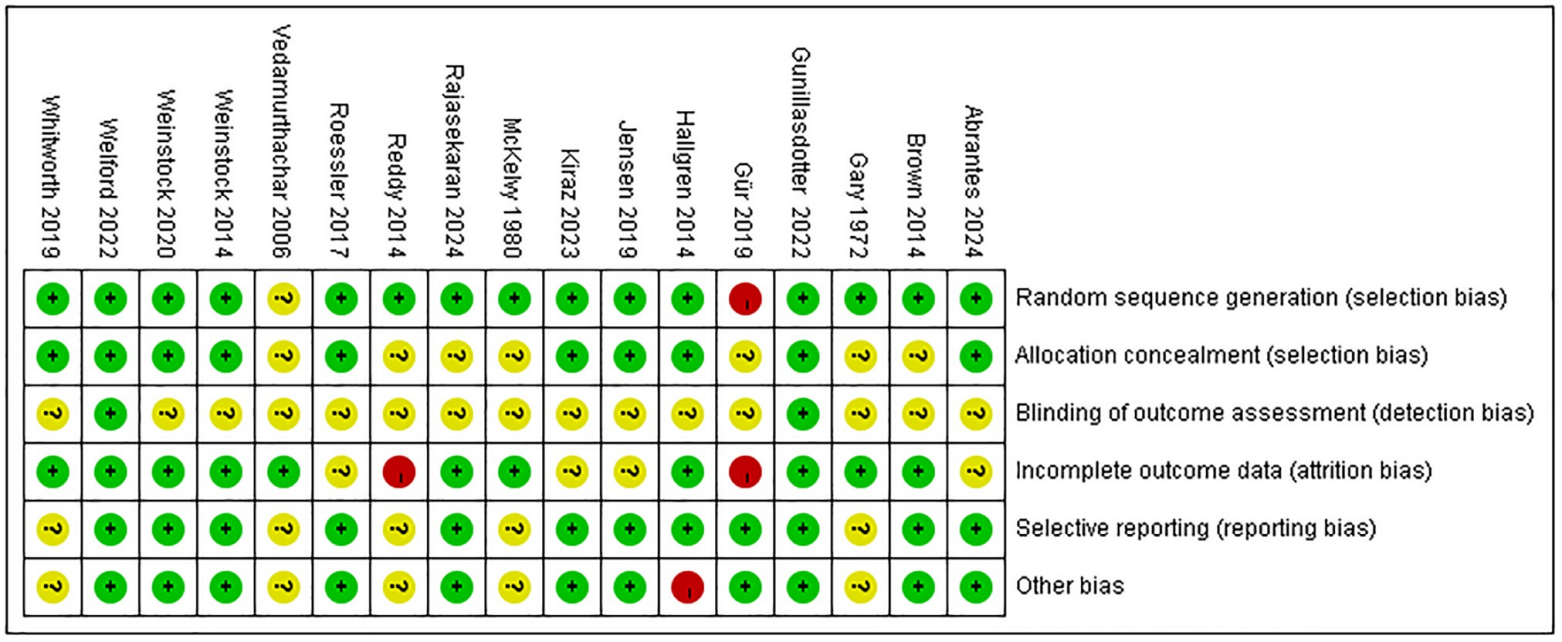
Summary graph of bias in the included literature.

The results of the risk of bias evaluation of the included studies are shown in Figs 2 and 3. Two researchers evaluated the risk of bias of the 17 included papers via the Cochrane risk of bias assessment tool (ROB2) in five dimensions.

Concerns regarding the randomization process were noted in 3 (18%) studies, and 1 was rated high ROB due to problems with the random assignment methodology. Due to the difficulty of implementing blinding as the intervention in this study was physical activity, 9 (53%) studies had problems with deviations from intended interventions. Two studies did not clearly report data completeness, and one was rated as high ROB due to a high subject dropout rate. Concerns regarding the selection of the reported result were raised in 1 (6%) studies. Overall, the risk of bias judgment indicated high ROB in 2 (12%) studies and low ROB in 7 (41%) studies. Overall, we considered number of drinks per day, anxiety, and depression to be at high risk of bias among the outcome indicators. Number of drinks per week, AUDIT, maximal oxygen uptake, resting heart rate, and stress were judged to be of some concern (S4 Table in S1 Appendix).

### Meta-analysis

In this study, three outcome indicators, daily alcohol consumption, weekly alcohol consumption, and the AUDIT test, were selected to determine the effect of alcohol dependence in AUD patients with exercise intervention; and five outcome indicators, VO^2^max, resting heart rate, anxiety level, depression level, and stress level, were selected to determine the effects of exercise interventions on the physical and mental states of AUD patients, and a meta-analysis of the results was performed (Table 1).

**Table 1:** Meta-analysis results.

Analysis	Number of ES	Number of participants	Meta-analysis			p Value	Heterogeneity		Publication bias
			SMD	95%CI					Egger bias p value
				Lower limit	Upper limit		I^2^	p Value	
NDPD	6	223	-0.661	-1.033	-0.288	0.001	53.5%	0.057	0.88
NDPW	5	233	-0.14	-0.224	0.656	0.374	33.9%	0.196	0.195
AUDIT	5	193	-0.36	-0.62	-0.1	0.007	0.0%	0.715	0.989
VO^2^_max_	5	197	0.406	0.116	0.697	0.006	12.7%	0.333	0.451
RHR	2	68	-0.863	-1.438	-0.288	0.003	14.5%	0.279	-
Anxiety	7	312	-0.791	-1.369	-0.213	0.007	84.2%	**< 0.001**	0.586
Depression	8	422	-0.86	-1.409	-0.31	0.002	87%	**< 0.001**	0.17
PSS	2	119	-2.127	-3.91	-0.344	0.019	93%	**< 0.001**	-

ES: Effect size; SMD: Standardized mean difference; CI: Confidence interval; NDPD: Number of drinks per day; NDPW: Number of drinks per week; AUDIT: Alcohol Use Disorders Identification Test; VO^2^max: Maximal oxygen uptake; RHA: Resting heart rate; PSS: Perceived Stress Scale.

### The effect of exercise on alcohol dependence in patients with AUD

#### Effect of exercise on number of drinks per day

**Fig 4:**
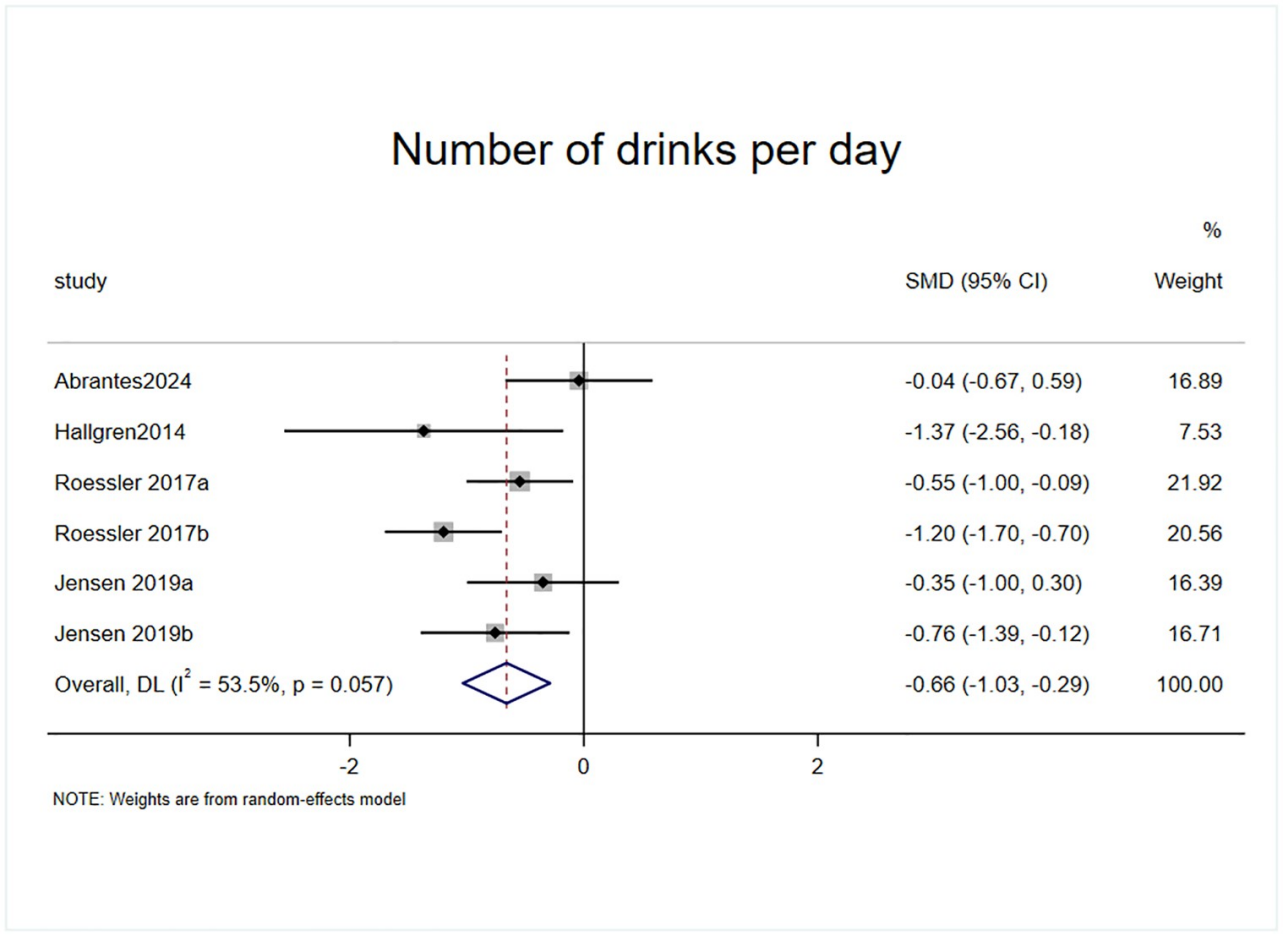
Effect of exercise on number of drinks per day.

Daily alcohol consumption was included as an outcome indicator in 4 randomized controlled trials, of which two studies had two experimental groups with a total of 6 effect sizes, containing a sample of 223 cases (Fig 4). The results of the heterogeneity test (I^2^ = 53.5%, p = 0.057) revealed that exercise intervention had a significant effect on reducing daily alcohol consumption in patients with AUD compared with the control group (SMD = -0.661, 95% CI = -1.033, -0.288, p = 0.001).

#### Effect of exercise on the number of drinks per week

**Fig 5:**
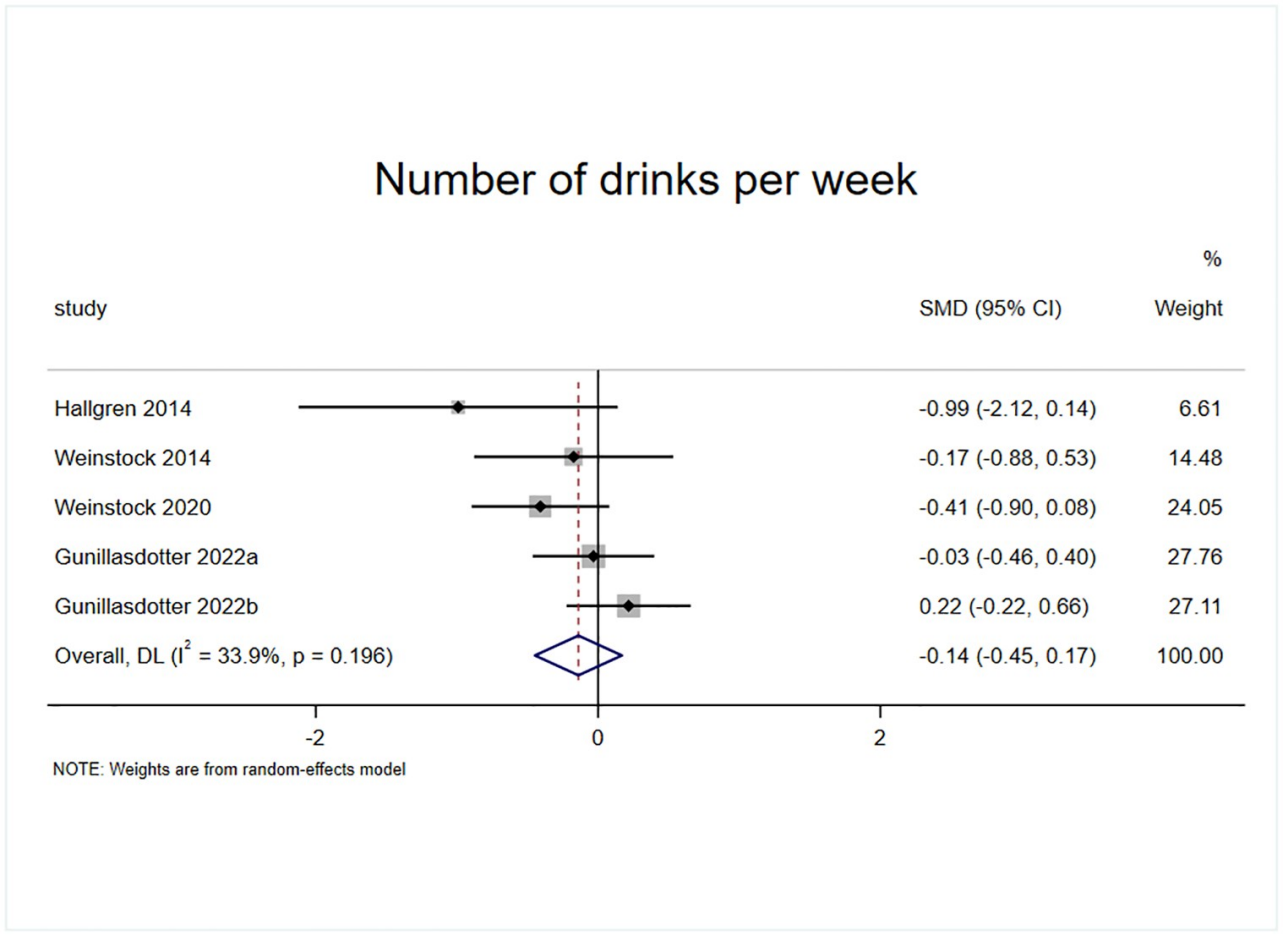
Effect of exercise on the number of drinks per week.

Four randomized controlled trials included weekly alcohol consumption as an outcome indicator, of which one study had two experimental groups with a total of five effect sizes, containing a sample of 233 cases (Fig 5). The results of the heterogeneity test (I^2^ = 33.9% p = 0.196) revealed that exercise intervention had no significant effect on weekly alcohol consumption in patients with AUD compared with the control group (SMD = -0.14, 95% CI = -0.224, -0.656, p = 0.374).

#### Effect of exercise on AUDIT scores

**Fig 6:**
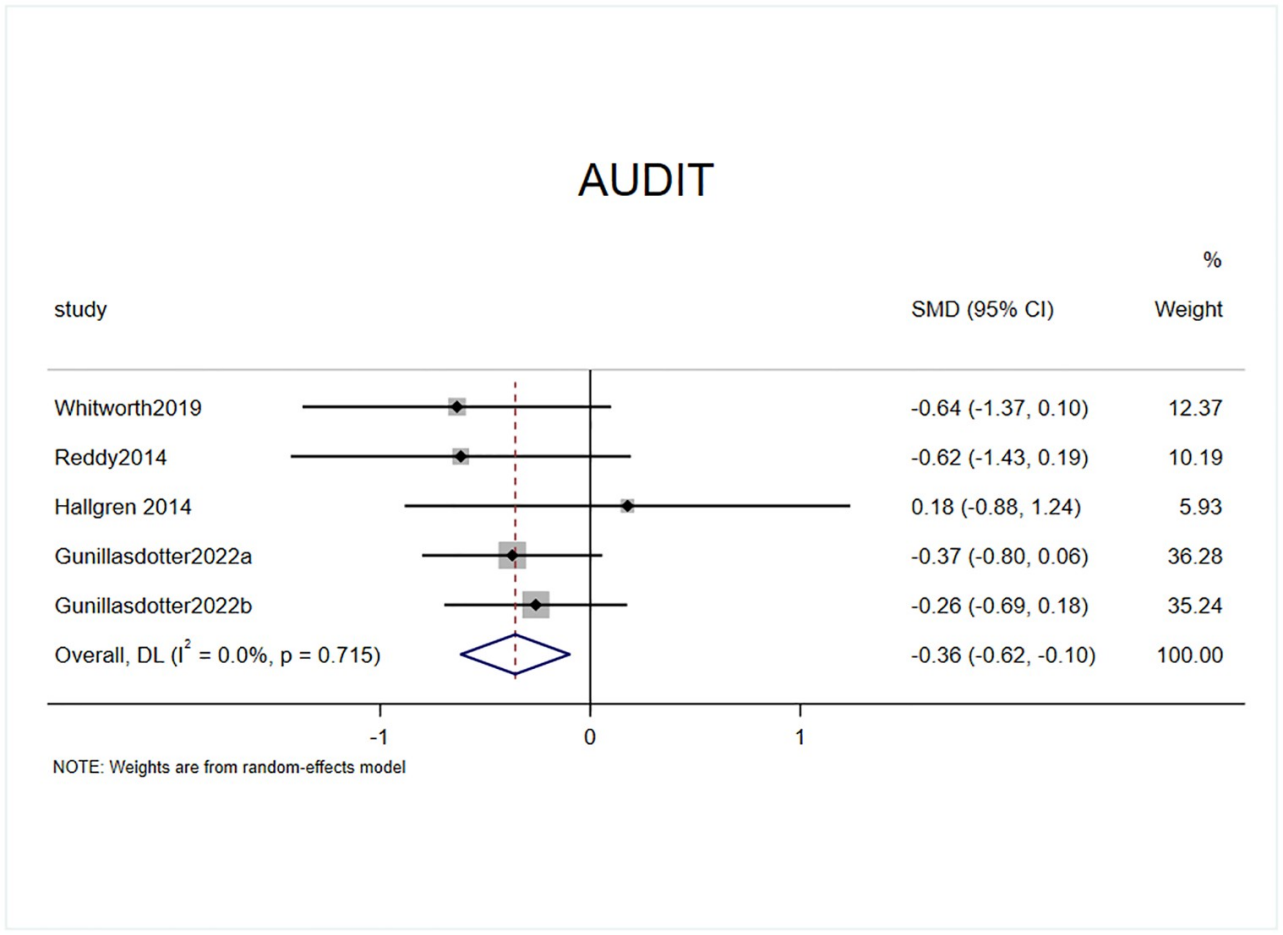
Effect of exercise on AUDIT scores.

Four randomized controlled trials included AUDIT ratings among the outcome indicators, and one of these studies had two experimental groups with a total of five effect sizes, containing a sample of 193 cases (Fig 6). The results by the heterogeneity test (I^2^ = 38.8%, p = 0.715) revealed that exercise intervention had a significant effect on the reduction in AUDIT score in patients with AUD compared with the control group (SMD = -0.36, 95% CI = -0.62, -0.1, p = 0.007).

### The effects of exercise on the physical and mental states of patients with AUD

#### Effect of exercise on VO^2^max

**Fig 7:**
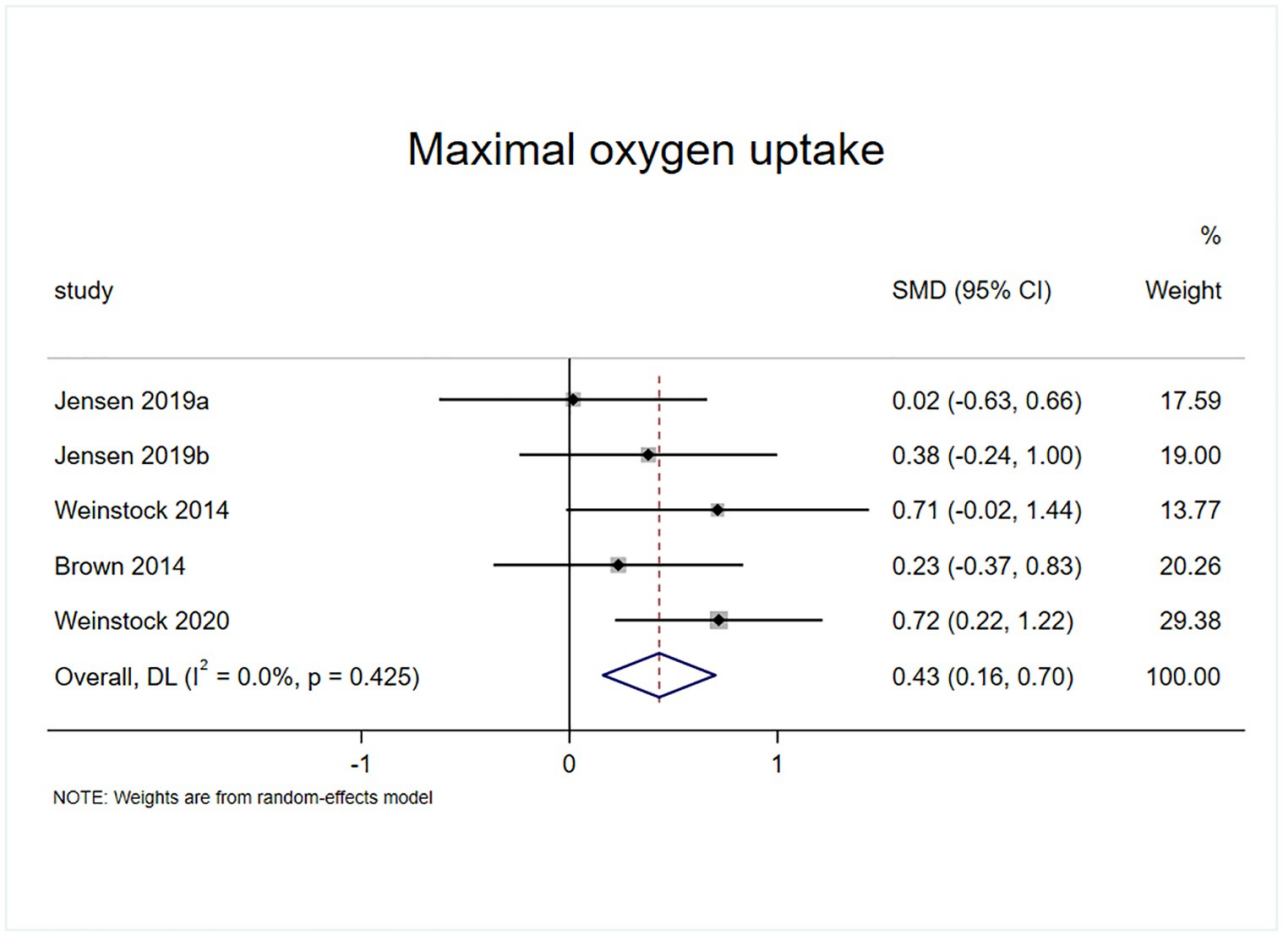
Effect of exercise on maximal oxygen uptake.

Four randomized controlled trials included VO^2^max as an outcome indicator, among which study 1 had two experimental groups with five effect sizes, containing a sample of 197 cases (Fig 7). The results of the heterogeneity test (I^2^ = 12.7%, p = 0.333) revealed that exercise intervention significantly improved VO^2^max in patients with AUD compared with the control group (SMD = 0.406, 95% CI = 00.116, 0.697, p = 0.006).

#### Effect of exercise on resting heart rate

**Fig 8:**
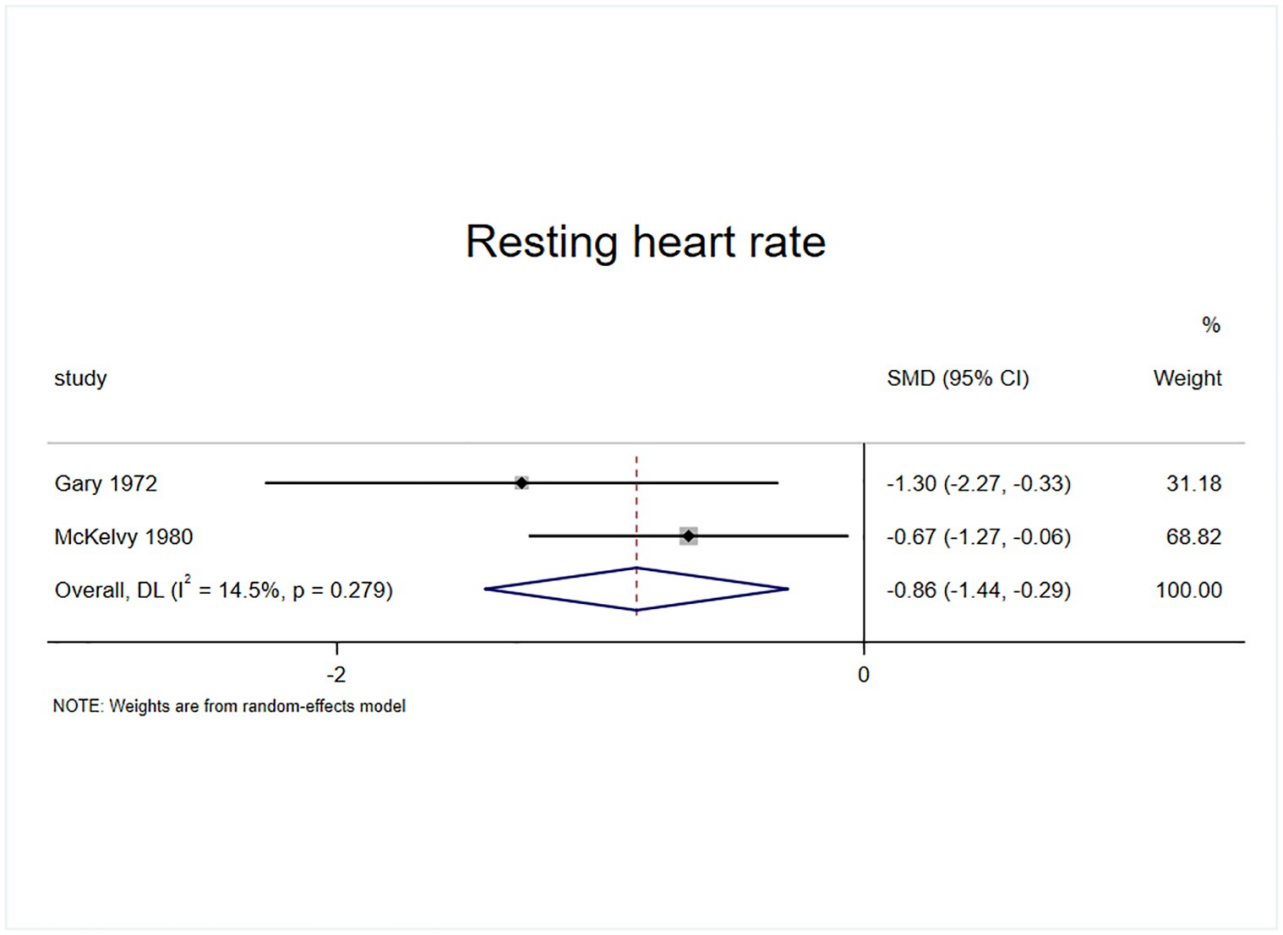
Effect of exercise on resting heart rate.

The resting heart rate was included as an outcome indicator in 2 randomized controlled trials with 2 effect sizes and a sample of 68 cases (Fig 8). The results of the heterogeneity test (I^2^ = 14.5%, p = 0.279) revealed that exercise intervention significantly improved the resting heart rate in patients with AUD compared with the control group (SMD = -0.863, 95% CI = -1.438, -0.2288, p = 0.003).

#### Effect of exercise on anxiety

**Fig 9:**
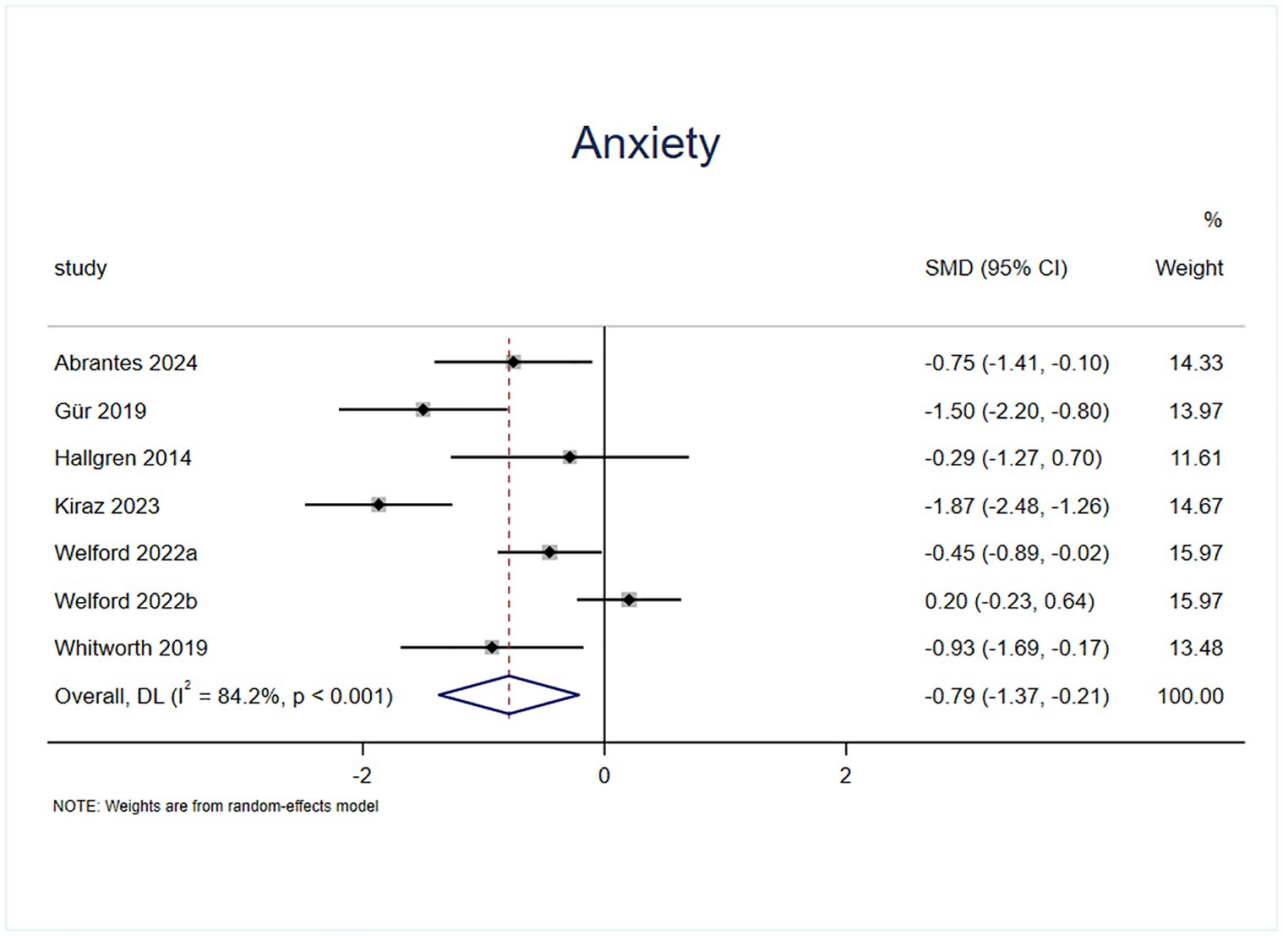
Effect of exercise on anxiety.

Anxiety status was included in the outcome indicators of six randomized controlled trials, of which one study had two experimental groups with a total of seven effect sizes, containing a sample of 312 cases (Fig 9). The results of the heterogeneity test (I^2^ = 84.2%, p = 0.00) revealed that, compared with the control intervention, the exercise intervention significantly improved anxiety in patients with AUD (SMD = -0.791, 95% CI = -1.369, -0.213, p = 0.007).

#### Effect of exercise on depression

**Fig 10:**
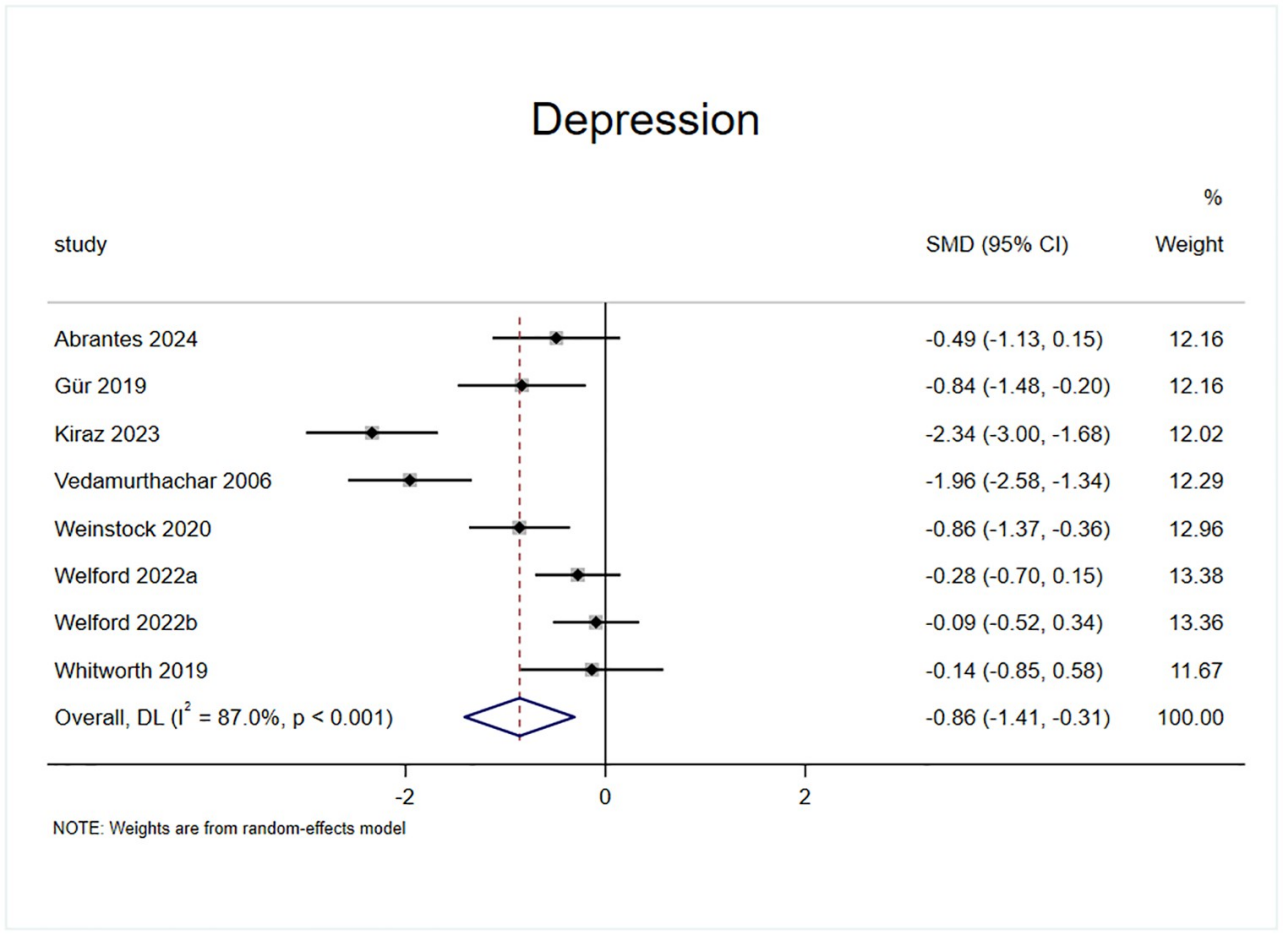
Effect of exercise on depression.

Depressive status was included in the outcome indicators of seven randomized controlled trials, of which eleven studies had two experimental groups with a total of eight effect sizes, comprising a sample of 422 cases (Fig 10). Analysis of the results of the heterogeneity test (I^2^ = 87%, p = 0.00) revealed that exercise intervention had a significant effect on reducing daily alcohol consumption in patients with AUD compared with the control group (SMD = -0.86, 95% CI = -1.409, -0.31, p = 0.002).

#### Effect of exercise on stress

**Fig 11:**
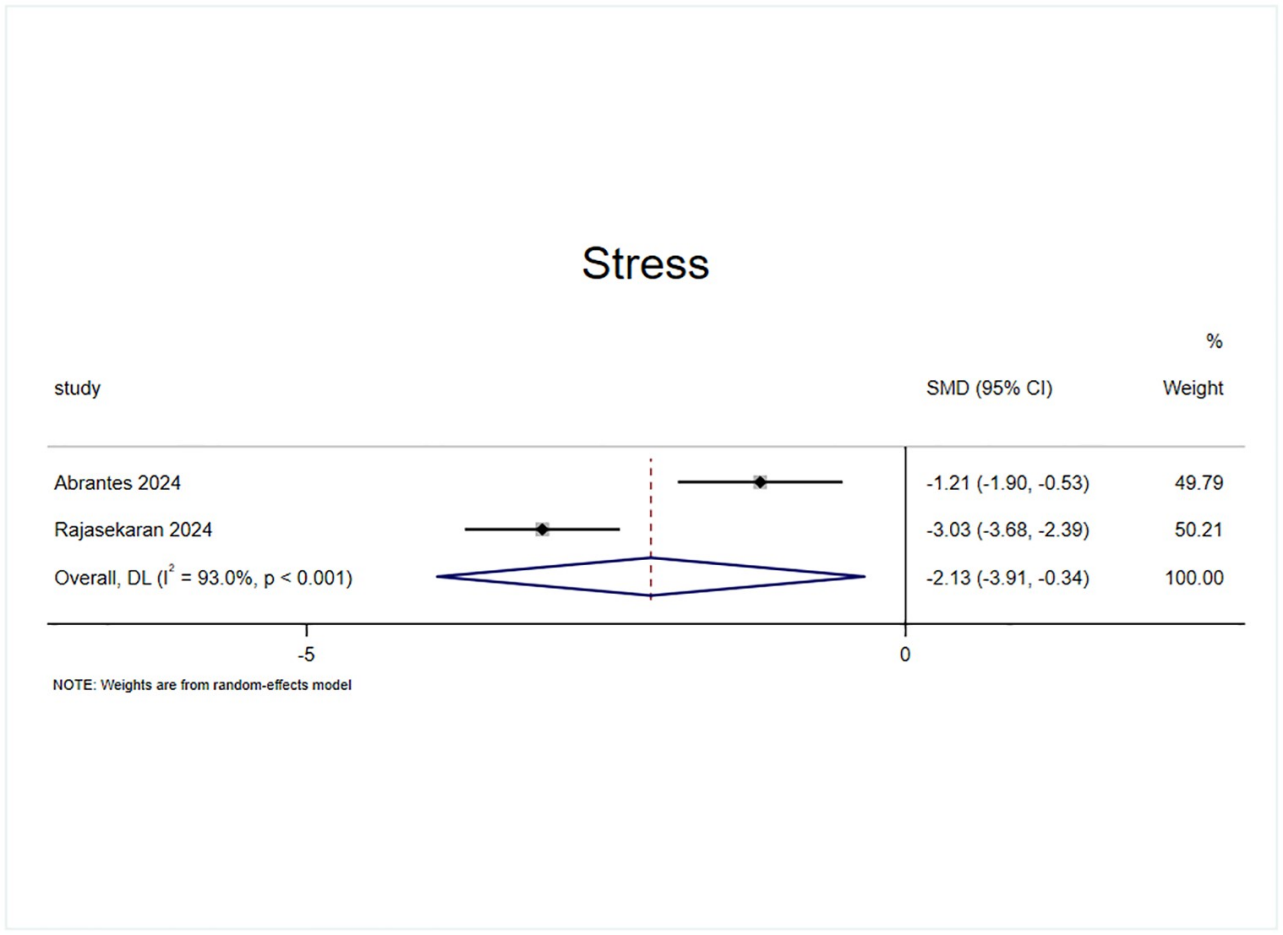
Effect of exercise on stress.

Stress status was included in the outcome indicators of the 2 randomized controlled trials with 2 effect sizes, which included a sample of 119 cases (Fig 11). The results of the heterogeneity test (I^2^ = 93%, p = 0.00) revealed that, compared with the control intervention, he exercise intervention significantly improved the stress status of patients with AUD (SMD = -2.127, 95% CI = -3.91, -0.344, p = 0.019).

### Publication bias

Egger’s test revealed no publication bias for daily alcohol consumption (p = 0.88), weekly alcohol consumption (p = 0.195), AUDIT (p = 0.989), VO^2^max (p = 0.303), anxiety (p = 0.586), or depression (p = 0.17), as the effect sizes of the reported resting heart rate and stress indicators were too small to perform Egger’s test could not be performed because of the small effect sizes reported for the resting heart rate and stress.

### GRADE assessment

The GRADE assessment showed (S5 Table in S1 Appendix) that the outcome metrics of Number of drinks per day, anxiety, and stress were rated Very low, and Number of drinks per week, AUDIT, maximal oxygen uptake, resting heart rate, and depression were rated Low.

### Subgroup analysis

Subgroup analysis revealed that there were no significant differences in the effects of different types of exercise, exercise intensity, duration of single intervention, exercise cycle or exercise format, such as daily alcohol consumption, weekly alcohol consumption, AUDIT score, VO^2^max, and depression level (P > 0.05, Table 2), on AUD patients. The subgroup analysis revealed significant differences in the effects of different types of exercise and intervention periods on the anxiety status of AUD patients (P < 0.05, Table 2), with the most effective type of exercise being mixed exercise, with an effect size of -1.709, and the most effective intervention period being 12 weeks or less, with an effect size of -1.221. There were no significant differences in the effects of exercise intensity, duration of a single intervention, or form of exercise on the anxiety status of AUD patients (P > 0.05, Table 2). Subgroup analyses could not be performed because the reported resting heart rate and stress index effects were too small.

**Table 2:** Subgroup analysis evaluating the effects of exercise on alcohol dependence and physical and mental status.

Analysis	No. of ES	Meta-Analytic Effect Size			Total Between		Heterogeneity
		SMD	95% CI				
			Lower limit	Upper limit	Q (df)	P Value	I^2^
Number of drinks per day							
Exercise type		-0.619	-0.963	-0.275	1.43 (1)	0.232	
Aerobic	5	-0.603	-0.990	-0.217			57.2%
Yoga	1	-1.368	-2.559	-0.176			-
Exercise intensity		-0.382	-0.791	0.028	1.69 (1)	0.193	
High	2	-0.558	-1.012	-0.103			0%
low	1	-0.042	-0.672	0.588			-
Length of a single workout		-0.674	-1.141	-0.206	1.55 (1)	0.213	
30–60 min	2	-0.558	-1.012	-0.103			0%
>60 min	1	-1.368	-2.559	-0.176			-
Exercise cycle		-0.619	-0.963	-0.275	1.43 (1)	0.205	
12 weeks or less	1	-1.368	-2.559	-0.176			-
>12 weeks	5	-0.603	-0.990	-0.217			57.2%
Group or not		-0.619	-0.963	-0.275	0.09 (1)	0.763	
Group	3	-0.567	-0.949	-0.185			7.9%
Individual	3	-0.661	-1.033	-0.288			74.9%
Number of drinks per weeks							
Exercise type		-0.140	-0.447	0.168	0.27 (1)	0.602	
Aerobic	3	-0.100	-0.510	0.310			43.8%
Yoga	2	-0.360	-1.260	0.530			58.6%
Length of a single workout		-0.140	-0.447	0.168	5.13 (2)	0.077	
30 min or less	2	-0.333	-0.734	0.068			0%
30–60 min	2	0.089	-0.219	0.397			0%
>60 min	1	-0.991	-2.122	0.139			-
Exercise cycle		-0.140	-0.447	0.168	0.86 (1)	0.354	
12 weeks or less	2	-0.460	-1.220	0.310			30.9%
>12 weeks	3	-0.060	-0.410	0.280			42.8%
AUDIT							
Exercise type		-0.357	-0.615	-0.099	0.74 (2)	0.69	
Aerobic	1	-0.260	-0.695	0.176			-
Resistance	1	-0.635	-1.370	0.100			-
Yoga	3	-0.357	-0.714	0.00			0%
Exercise intensity		-0.363	-0.646	-0.081	0.62 (1)	0.433	
High	1	-0.635	-1.370	0.100			-
Moderate	2	-0.316	-0.622	-0.011			0%
Length of a single workout		-0.357	-0.615	-0.099	0.03 (1)	0.872	
30–60 min	3	-0.363	-0.646	-0.081			0%
>60 min	2	-0.297	-1.060	0.466			26.4%
Exercise cycle		-0.328	-0.600	-0.055	0.00 (1)	0.988	
12 weeks or less	2	-0.323	-1.097	0.452			34.3%
>12 weeks	2	-0.316	-0.622	-0.011			0%
Group or not		-0.357	-0.615	-0.099	0.63 (1)	0.428	
Group	4	-0.318	-0.594	-0.042			0%
Individual	1	-0.635	-1.370	0.100			-
Vo^2^max							
Exercise intensity		0.406	0.116	0.697	1.81 (1)	0.179	
High	2	0.161	-0.285	0.606			0%
Moderate	3	0.554	0.194	0.913			9.7%
Length of a single workout		0.406	0.116	0.697	1.81 (1)	0.179	
30 min or less	3	0.554	0.194	0.913			9.7%
30–60 min	2	0.161	-0.285	0.606			0%
Exercise cycle		0.406	0.116	0.697	0.98 (1)	0.321	
12 weeks or less	1	0.75	0.020	1.480			-
>12 weeks	4	0.346	0.026	0.697			17.2%
Group or not		0.406	0.116	0.697	0.1 (1)	0.755	
Group	4	0.423	0.052	0.794			32.6
Individual	1	0.309	-0.309	0.926			-
Anxiety							
Exercise type		-0.791	-1.369	-0.213	19.33 (3)	**< 0.001**	
Aerobic	2	-0.243	-1.179	-0.694			82.6%
Resistance	1	-0.931	-1.687	-0.175			-
Yoga	2	-0.428	-0.822	-0.033			0%
Mixed	2	-1.709	-2.168	-1.250			0%
Exercise intensity		-0.884	-1.647	-0.121	0.01 (1)	0.931	
High	1	-0.931	-1.687	-0.175			-
Moderate	4	-0.878	-1.796	0.039			91.8%
Length of a single workout		-0.799	-1.475	-0.123	0.88 (1)	0.348	
30–60 min	5	-0.884	-1.647	-0.121			89.3%
>60 min	1	-0.287	-1.273	0.699			-
Exercise cycle		-0.791	-1.369	-0.213	4.62 (1)	**0.032**	
12 weeks or less	4	-1.221	-1.853	-0.588			65.1%
>12 weeks	3	-0.302	-0.851	0.248			73%
Group or not		-0.791	-1.369	-0.213	0.001 (1)	0.909	
Group	5	-0.775	-1.565	0.014			89.2%
Individual	2	-0.83	-1.369	-0.336			0%
Depression							
Exercise type		-0.86	-1.409	-0.310	3.61 (3)	0.307	
Aerobic	3	-0.466	-0.948	0.015			61.3%
Resistance	1	-0.136	-0.853	0.580			-
Yoga	2	-1.101	-2.747	0.545			94.8%
Mixed	2	-1.584	-3.053	-0.115			87%
Exercise intensity		-0.86	-1.409	-0.310	2.85 (2)	0.241	
High	1	-0.136	-0.853	0.580			-
Moderate	5	-0.851	-1.538	-0.164			88.7%
low	2	-1.226	-2.662	0.209			90.4%
Length of a single workout		-0.913	-1.533	-0.292	1.03 (1)	0.311	
30 min or less	2	-1.393	-2.467	-0.319			86.2%
30–60 min	5	-0.718	-1.458	0.022			88.9%
Exercise cycle		-0.86	-1.409	-0.310	3.13 (1)	0.077	
12 weeks or less	4	-1.324	-2.286	-0.361			88.3%
>12 weeks	4	-0.405	-0.737	-0.072			45.6%
Group or not		-0.86	-1.409	-0.310	2.69 (1)	0.101	
Group	6	-1.034	-1.721	-0.348			90.1%
Individual	2	-0.334	-0.811	0.143			0%

ES = Effect size.

### Best evidence synthesis

For the primary outcome indicator (alcohol craving), we conclude that the available research evidence for the effect of exercise interventions on alcohol craving in patients with AUD is only moderate. Nine randomized controlled trials reported on the outcome indicator of alcohol cravings; four studies with six effect sizes reported a significant reduction in daily alcohol consumption in patients with AUD, and four studies with five effect sizes reported a significant reduction in the AUDIT score in patients with AUD. Seven of the nine randomized controlled trials were of high quality, two were of low quality, and only 1 of these randomized controlled trials used blinded assessment.

For the secondary outcome indicators (physical and mental state), we conclude that there is a strong level of evidence for the effects of exercise interventions on physical and mental states in patients with AUD as demonstrated by existing studies. Thirteen randomized controlled trials reported outcome measures of physical and mental status before and after exercise intervention in patients with AUD, and the results revealed that exercise intervention had significant effects on VO^2^max, resting heart rate, anxiety, depression, and stress in patients with AUD. Ten of the 9 randomized controlled trials were of high quality, 2 were of moderate quality, only 1 was of low quality, and 1 of these studies used blinded assessment of outcome. Assessment. In a subgroup analysis of the physical and psychological status of patients with AUD, there was a significant difference between the type of exercise and the intervention period on the anxiety level of patients with alcohol use disorders, with the optimal type of training and intervention period being a mixture of exercise types and an intervention period of 12 weeks or less, respectively.

## Discussion

Among the 17 RCTs included in this study, 1905 patients with alcohol use disorders participated in the experiments; these patients were divided into 21 control groups (4 of these RCTs were divided into 2 experimental groups [26–28,40]) and 17 experimental groups. The changes in daily alcohol consumption, AUDIT score, VO^2^max, resting heart rate, anxiety state, depression state, stress level and other indices were significantly different between the experimental group and the control group; only the index of weekly alcohol consumption did not reach statistical significance, indicating that exercise has a good intervention effect on alcohol dependence and the physical and mental states of AUD patients. In addition, a subgroup analysis of the included studies was performed according to the outcome indicators (type of exercise, intensity, duration of a single exercise session, intervention period, and form of exercise), which revealed that the type of exercise and the intervention period significantly affected the anxiety level of the patients with alcohol use (p<0.05), thus indicating that the better exercise intervention for their anxiety was mixed exercise, and the more appropriate intervention period was 12 weeks or less; although most of the subgroups did not present statistically significant results, the results should be due to the small number of studies included in this study. To assess risk of bias, we used the revised Cochrane RoB 2, which I assessed for the primary outcome and for all studies. We also assessed the quality (or credibility) of the evidence using the GRADE method. Overall, this study provides new evidence to assist in the treatment of patients with alcohol use disorders, validating the findings of the study of the Lardier D T team [24], which demonstrated that exercise interventions can be used as adjunctive medical treatments for alcohol dependence.

Daily and weekly alcohol consumption before and after the exercise intervention were the most direct indicators of alcohol use in patients with AUD. After combining the effect size analyses, the present study concluded that the change in number of drinks per day in the experimental group (SMD = -0.66) was significantly better than that in the control group [25–27,36], and the test of heterogeneity (I^2^ = 53.5%, p = 0.057) indicated that exercise intervention had some heterogeneity in reducing number of drinks per day. After excluding one effect size by sensitivity analysis [36] (SMD = -0.76), the heterogeneity was reduced to (I^2^ = 33%, p = 0.22), which was significantly lower, and the effect size was still statistically significant. Suggesting that the exercise intervention had a stronger effect on the reduction in number of drinks per day in patients with AUD, which validated the findings of the Lardier D T et al. study [24]. However, changes in weekly alcohol consumption did not reach statistical significance (p = 0.374) [25,28,41,43], and we excluded one of the low-quality papers and analyzed it again, and the results also did not reach statistical significance (p = 0.599); the age of the analyzed subjects who reported daily alcohol consumption was concentrated in the 40–50 years age range, and the age of the subjects who reported weekly alcohol consumption was concentrated in the 20–35 and 50–55 years age ranges, which may be the result of inconsistent results due to differences in the subjects’ ages; we also considered that the small effect size analyzed may be the reason for the inconsistency of the results. For the determination of alcohol dependence we also selected the Alcohol Use Disorder Identification Test (AUDIT), a screening tool for alcohol use disorders [47], and analyzed the results (SMD = -0.3), indicating that the exercise intervention had a moderate effect on the improvement in the AUDIT score. The test of heterogeneity (I^2^ = 38.8%, p = 0.715) indicated that the exercise intervention had good consistency in reducing AUDIT scores, and the above results provide evidence for exercise intervention for alcohol dependence in patients with AUD.

The improvement in the physical fitness of AUD patients by exercise was reflected by VO^2^max and the resting heart rate. Regarding VO^2^max, there was a significant difference between the experimental group and the control group in the improvement in VO^2^max (SMD = 0.406), indicating that the exercise intervention had a moderate effect on the improvement in VO^2^max in patients with AUD, and the test of heterogeneity (I^2^ = 12.7%, p = 0.333) indicated that there was good consistency between the studies. With respect to the resting heart rate, there was a significant difference in the improvement in the resting heart rate between the experimental group and the control group (SMD = -0.863), and the test of heterogeneity (I^2^ = 14.5%, p = 0.279) indicated that there was good agreement between the studies, and that the exercise intervention had a strong effect on the improvement in the resting heart rate in patients with AUD.

The underlying mechanism of the effect of exercise intervention on alcohol dependence may involve a reduction in psychological stress, an improvement in mental state, and an influence on the dopamine system [14,48,49]; therefore, in the present study, three indices related to the mental state, namely, anxiety, depression, and stress, were selected to validate the effect of exercise intervention in patients with AUD. The experiments included in this study on measuring anxiety states used measures such as the Generalized Anxiety Disorder—7 Item Scale (GAD-7) [50], Beck Anxiety Inventory (BAI) [51], Hospital Anxiety and Depression (HAD) [52], Hamilton anxiety scale (HAM-A) [53], State-Trait Anxiety Inventory (STAI) [54] and other different scales. The results showed that the anxiety state of the experimental group was significantly improved compared with the control group (SMD = -0.791), indicating that the exercise intervention was effective in alleviating the anxiety state of AUD patients, verifying the view of Hallgren M et al [23]. The heterogeneity test showed (I^2^ = 84.2%, p = 0.00) that there was a high degree of heterogeneity between the studies, and after excluding 2 effect sizes by sensitivity analysis (SMD = -0.76) [40,45], the heterogeneity was significantly reduced (I^2^ = 43%, p = 0.14). The effect size was still statistically significant, suggesting that exercise interventions have a strong effect on the reduction of anxiety states in patients with AUD. The experiments included in this study on measuring depressive states used different scales, such as the Behavioral Activation for Depression Scale (BADS) [55], Beck Depression Inventory (BDI) [56], Hamilton Depression Scale (HAM-D) [57], Center for the Epidemiologic Studies of Depression Short Form (CESD) [58] and other scales. The results revealed that the reduction in anxiety in the experimental group was significantly greater than that in the control group (SMD = -0.8), which verified the findings of the study of GÜR F et al [22], indicating that the exercise intervention had a strong effect on the reduction in anxiety in patients with AUD. The heterogeneity test (I^2^ = 87%, p = 0.00) revealed a high degree of heterogeneity between the studies after the exclusion of the 2 effect sizes through the sensitivity analysis [38,45]. The result was SMD = -0.4, and the heterogeneity was reduced to I^2^ = 34%, p = 0.18 Heterogeneity was significantly reduced and the effect sizes were still statistically significant, suggesting that the exercise intervention had a moderate effect on the alleviation of depressive states in patients with AUD. Regarding the measurement of stress levels, two effect sizes were reported in the included experiments [36,39], both of which were measured via the Perceived Stress Scale (PSS) [59], and the results revealed that the reduction in anxiety states in the experimental group was significantly greater than that in the control group (SMD = -2.12), indicating that the exercise intervention had a stronger effect on the reduction in anxiety states of the patients with AUD. A heterogeneity test (I^2^ = 93%, p = 0.00) There was a high degree of heterogeneity between the studies, but sensitivity analyses could not be performed because the effect size was too small to allow sensitivity analyses to be performed; thus the present study provided low evidence of exercise intervention for stress states.

Regarding the type of exercise, this study revealed that since most AUD patients have poor physical functioning and lower-than-average physical fitness due to chronic excessive alcohol consumption and a lack of exercise [60], the choice of exercise intervention should be individualized. With respect to the type of exercise for AUD patients, yoga was chosen as an exercise intervention in 6 of the 17 RCTs included in this study [25,28,30,38–40], accounting for 35% of the total number of RCTs. Yoga is a mind-body exercise that integrates breathing and mental training while practicing physical postures [61,62], Kuppili and Sarkar et al.’s studies explored the effectiveness of yoga in the treatment of substance use disorders (SUD) [63–66], in which they demonstrated the efficacy of yoga as an intervention for AUD. Although the subgroup analyses in this study did not reveal that the effects of yoga exercise on the outcome indicators were significantly different from those of other types of exercise, yoga exercise significantly improved daily alcohol consumption (SMD = -1.368), AUDIT (SMD = -0.357), and anxiety (SMD = -0.428), combined with the low intensity of yoga exercise and its favorable effect on psychological state intervention. This study demonstrated that yoga exercise is a good intervention to complement AUD treatment.

In this study, on the basis of the results of previous studies, we selected several outcome indicators to test alcohol dependence, added randomized controlled trials with more recent years, and verified the conclusions of previous studies. To further verify the effectiveness of the exercise intervention in enhancing the physical and mental states of AUD patients, we selected five outcome indicators of physical and mental states to be analyzed individually. At the same time, there are several limitations. First, some of the studies included in this meta-analysis did not specify the method of randomization, and only two randomized controlled trials reported blinding in the experiments. This ambiguity in study design reduces the quality of the literature and may affect the evaluation of intervention effects. Second, the included studies were published in English only and did not include studies published in other languages. The current status of alcohol addiction varies according to cultural context, and conclusions should be extrapolated with caution, which may lead to biased results in the analysis. Third, the outcome indicator of alcohol consumption used in this study was measured, and there may be a comparability issue due to the inconsistency in the unit of measurement of outcomes across studies. For this reason, we used standardized mean difference joint effect sizes to minimize the effect of differences.

## Conclusions

This systematic review provides evidence for the adjunctive treatment of patients with alcohol use disorder (AUD) through exercise intervention. The synthesized evidence revealed that exercise intervention significantly improved alcohol dependence and physical and mental status in the experimental group, and the changes in two of the three outcome indicators of alcohol dependence (daily alcohol consumption, AUDIT) were statistically significant, indicating that exercise has an effective intervention effect on alcohol dependence in AUD patients and can be used as an adjunctive treatment for AUD. The changes in the five outcome indicators of physical and mental status were statistically significant, indicating that exercise can effectively improve the VO^2^max, resting heart rate, anxiety, depression, and stress level of AUD patients, which proves that exercise can be an adjunctive means of improving the quality of life and reducing the relapse rate of AUD patients in conjunction with clinical treatments.

In this study, subgroup analyses of all outcome indicators were performed separately, but only the effects of different exercise types and intervention cycles on anxiety status were statistically significant. This study was unable to prove whether there were differences in the effects of different exercise types, exercise intensities, single intervention times, intervention cycles, and forms of exercise on alcohol dependence in patients with AUD. Therefore, this study calls for future studies to carefully differentiate between the types of exercise and exercise intensity and to analyze the effects of exercise of different natures on alcohol dependence more deeply to develop the most suitable exercise prescriptions for the clinical treatment of AUD according to different populations.

## Supporting information

S1 Checklist PRISMA 2020 checklist. (DOCX)

S1 Appendix (DOCX)

S1 Dataset (XLSX)
