# Supplementary material for: Effectiveness of exercise intervention in improving physical and mental status of patients with alcohol use disorders: A systematic review and meta-analysis
Source: PLoS One. 2024 Oct 30;19(10):e0311166. doi: 10.1371/journal.pone.0311166 (PMC11524501; doi:10.1371/journal.pone.0311166)
Supplement: S1 Appendix — (DOCX) [file pone.0311166.s002.docx]

**S2** **Appendix**

**S1 Table.** **Search strategy.**

| PubMed (Retrieved June 2024) |
| --- |
| ((("Exercise"[Mesh]) OR ((((((training[Title/Abstract]) OR (physical activity[Title/Abstract])) OR (aerobic training[Title/Abstract])) OR (resistance training[Title/Abstract])) OR (aerobic combined with resistance training[Title/Abstract])) OR (sport[Title/Abstract]))) AND (("Alcoholism"[Mesh]) OR (((((((((alcohol related[Title/Abstract]) OR (alcohol dependence[Title/Abstract])) OR (alcohol use disorder[Title/Abstract])) OR (hazardous drinking[Title/Abstract])) OR (harmful drinking[Title/Abstract])) OR (alcohol abuse[Title/Abstract])) OR (alcohol addiction[Title/Abstract])) OR (binge drinking[Title/Abstract])) OR (heavy drinking[Title/Abstract])))) AND ((((randomized controlled trial[Title/Abstract]) OR (randomized[Title/Abstract])) OR (placebo[Title/Abstract])) OR (RCT[Title/Abstract])) |
| Web of Science (Retrieved June 2024) |
| TS=(exercise OR training OR physical activity OR aerobic training OR resistance training OR aerobic combined with resistance training OR sport) AND TS=(alcoholism OR alcohol related OR alcohol dependence OR alcohol use disorder OR hazardous drinking OR harmful drinking OR alcohol abuse OR alcohol addiction OR binge drinking OR heavy drinking) AND TS=(randomized controlled trial OR randomized OR placebo OR RCT) |
| Cochrane Library (Retrieved June 2024) |
| (Exercise[Mesh]) or (training):ti,ab,kw or (physical activity):ti,ab,kw or (aerobic training):ti,ab,kw or (resistance training):ti,ab,kw or (aerobic combined with resistance training):ti,ab,kw or (sport):ti,ab,kw and (Alcoholism[Mesh]) or (alcohol related):ti,ab,kw or (alcohol dependence):ti,ab,kw or (alcohol use disorder):ti,ab,kw or (hazardous drinking):ti,ab,kw or (harmful drinking):ti,ab,kw or (alcohol abuse):ti,ab,kw or (alcohol addiction):ti,ab,kw or (binge drinking):ti,ab,kw or (heavy drinking):ti,ab,kw and (randomized controlled trial):ti,ab,kw or (randomized):ti,ab,kw or (placebo):ti,ab,kw or (RCT):ti,ab,kw |
| EBSCO (Retrieved June 2024) |
| (AB exercise OR AB training OR AB physical activity OR AB aerobic training OR AB resistance training OR AB aerobic combined with resistance training OR AB sport) AND (AB alcoholism OR AB alcohol related OR AB alcohol dependence OR AB alcohol use disorder OR AB hazardous drinking OR AB harmful drinking OR AB alcohol abuse OR AB alcohol addiction OR AB binge drinking OR AB heavy drinking) AND(AB randomized controlled trial OR AB randomized OR AB placebo OR AB RCT) |
| Embase (Retrieved June 2024) |
| Exercise [Emtree term] or 'training':ab,ti or 'physical activity':ab,ti or 'aerobic training':ab,ti or 'resistance training':ab,ti or 'aerobic combined with resistance training':ab,ti or 'sport':ab,ti and Alcoholism [Emtree term] or 'alcohol related':ab,ti or 'alcohol dependence':ab,ti or 'alcohol use disorder':ab,ti or 'hazardous drinking':ab,ti or 'harmful drinking':ab,ti or 'alcohol abuse':ab,ti or 'alcohol addiction':ab,ti or 'binge drinking':ab,ti or 'heavy drinking':ab,ti and 'randomized controlled trial':ab,ti or 'randomized':ab,ti or 'placebo':ab,ti or 'RCT':ab,ti |

S2 Table. Detailed characteristics of the included studies.

| **Author/Year** | **nation** | **Sample size** | | | **Age** | | | **Type of intervention** | | | **strength** | **time** | **frequency** | **period** | **G or I** | **Outcome measurements** |
| --- | --- | --- | --- | --- | --- | --- | --- | --- | --- | --- | --- | --- | --- | --- | --- | --- |
|  |  | **Ta** | **Tb** | **C** | **Ta** | **Tb** | **C** | **Ta** | **Tb** | **C** |  |  |  |  |  |  |
| Abrantes 2024 | USA | 25 | - | 25 | 44.9±11.7 | - | 37.0±11.2 | LPA | - | HEC | low | - | - | 12 weeks | I | NDPD; Anxiety; Depression; PSS |
| Brown 2014 | USA | 26 | - | 23 | 43.46±11.5 | - | 45.39±9.99 | AE | - | BA-E | Moderate | 30 min | 3times-wk | 12 weeks | G | VO^2^max |
| Gary 1972 | USA | 10 | - | 10 | 38.9 | - | 45.1 | AE | - | TAU | low | - | 5times-wk | 4 weeks | G | RHR |
| Gunillasdotter 2022 | Greece | 46 | 49 | 49 | 54 ±11 | 54 ±13 | 53 ±12 | Yoga | AE | TAU | Moderate | 60 min | 3times-wk | 12 weeks | G | HDTW; AUDIT |
| Gür 2019 | Turkey | 20 | - | 20 | 46.85±12.07 | - | 44.33±10.2 | Mixed | - | NA | Moderate | 60 min | 3times-wk | 6 weeks | G | Anxiety; Depression |
| Hallgren 2014 | Sweden | 8 | - | 6 | - | - | - | Yoga+TUA | - | TAU | - | 90 min | 1times-wk | 10 weeks | G | NDPD; HDTW; AUDIT; Anxiety |
| Jensen 2019 | Denmark | 16 | 19 | 22 | 45.6±11.3 | 41.4±11.2 | 48.2±11.8 | AE (G) +TAU | AE (I) +TAU | TAU | High | 45 min | 2times-wk | 24 weeks | G/I | NDPD;  VO^2^max |
| Kiraz 2023 | Turkey | 30 | - | 30 | 26.50 ± 5.31 | - | 27.43 ± 5.19 | Mixed | - | TUA | Moderate | 60 min | 5times-wk | 3 weeks | G | Anxiety; Depression |
| McKelvy 1980 | USA | 31 | - | 17 | 23.7±5.0 | - | 24.0±4.2 | AE | - | NA | low | - | 5times-wk | 4 weeks | G | RHR |
| Rajasekaran 2024 | India | 40 | - | 40 | 41.64±6.12 | - | 43.48±6.59 | Yoga+TUA | - | TAU | - | 52 min | 3times-wk | 8 weeks | G | PSS |
| Reddy 2014 | USA | 20 | - | 18 | 45.5±12.1 | - | 43.2±12.9 | Yoga | - | NA | - | 75 min | - | - | G | AUDIT |
| Roessler 2017 | Denmark | 60 | 60 | 53 | 44.8±11.2 | 43.8±11.1 | 46.9 ±11. | AE  (G) | AE  (I) | NA | - | - | 2times-wk | 24 weeks | G-I | NDPD |
| Vedamurthachar 2006 | India | 30 | - | 30 | 35.6±8.07 | - | 37.77±7.34 | Yoga | - | NA | low | 30 min | 3times-wk | 2 weeks | G | Depression |
| Weinstock 2014 | USA | 16 | - | 15 | 20.1±1.2 | - | 21.0±1.3 | AE(CM+MET) | - | MET | Moderate | 15 min | 3times-wk | 8 weeks | I | HDTW;  VO^2^max ; |
| Weinstock 2020 | USA | 33 | - | 33 | 33.76±8 | - | 35.9±12 | AE(MI+CM) | - | MO | Moderate | 30 min | 3times-wk | 16 weeks | I | HDTW; VO^2^max; Depression; |
| Welford 2022 | Sweden | 46 | 49 | 45 | 54.2±13.4 | 53.9±10.6 | 52.9±11.5 | Yoga | AE | TAU | Moderate | 60 min | 3times-wk | 12 weeks | G | Anxiety; Depression; |
| Whitworth 2019 | USA | 15 | - | 15 | 27.67±5.95 | - | 30.53±8.66 | RE | - | HE | High | 30 min | 3times-wk | 3 weeks | G | AUDIT; Anxiety; Depression; |

T: experimental group; C: control group; M: male; F: female; NA: not available; LPA: lifestyle physical activity; HEC: health education contact; AE: aerobic; RE: resistance; BA-E：brief advice to exercise intervention; TAU: treatment as usual; NDPD: number of drinks per day; NDPW: number of drinks per weeks; AUDIT: alcohol use disorders identification test; VO^2^max: maximal oxygen uptake; RHR: resting heart rate; PSS: perceived stress scale; MET: motivational enhancement therapy; CM: contingency management; MI: motivational interviewing; MO: gym membership only; HE: health education

S3 Table. Exclusion of literature.

| Author/Year | Title | Reason for exclusion |
| --- | --- | --- |
| Brown 2008[1] | Aerobic Exercise for Alcohol Recovery Rationale, Program Description, and Preliminary Findings | Studies that did not align with theresearchtopic |
| Giesen 2016[2] | Effects of an Exercise Program on Physical Activity Level and Quality of Life in Patients with Severe Alcohol Dependence | Studies that did not align with theresearchtopic |
| Hallgren 2018[3] | Physical activity as treatment for alcohol use disorders (FitForChange): study protocol for a randomized controlled trial | Studies that did not align with theresearchtopic |
| Hovhannisyan 2020[4] | Evaluation of Very Integrated Program (VIP): Health promotion for patients with alcohol and drug addiction — A Randomized Trial | Studies that did not align with theresearchtopic |
| Piorkowski 1976[5] | Effect of Circuit Exercise Trainingon Physical Fitness of Alcoholic Patients | Studies that did not align with theresearchtopic |
| Roessler 2018[6] | Interpersonal problems of alcohol use disorder patients undergoing a physical exercise intervention – A randomised controlled trial | Studies that did not align with theresearchtopic |
| Sari 2019[7] | The effect of exercise as adjunctive treatment on quality of life for individuals with alcohol use disorders: a randomized controlled trial | Studies that did not align with theresearchtopic |
| Weinstock 2016[8] | Sedentary College Student Drinkers Can Start Exercising and Reduce Drinking After Intervention | Studies that did not align with theresearchtopic |
| Houghton 2017[9] | Effects of Exercise on Liver Fat and Metabolism in Alcohol Drinkers | Studies that did not align with theresearchtopic |
| Williams 2004[10] | The relationships of vigorous exercise, alcohol, and adiposity to low and high high-density lipoprotein-cholesterol levels | Studies that did not align with theresearchtopic |
| El-Sayed 2005[11] | Interaction between alcohol and exercise: physiological and haematological implications | Studies that did not align with theresearchtopic |
| Yokoyama 2009[12] | Associations between headache and stress, alcohol drinking, exercise, sleep, and comorbid health conditions in a Japanese population | Studies that did not align with theresearchtopic |
| Müller 2006[13] | Risk for exercise dependence, eating disorder pathology, alcohol use disorder and addictive behaviors among clients of fitness centers | Studies that did not align with theresearchtopic |
| Fadus 2019[14] | Adolescent Substance Use Disorder Treatment: an Update on Evidence-Based Strategies | Studies that did not align with theresearchtopic |
| Suter 2008[15] | The effect of exercise, alcohol or both combined on health and physical performance | Studies that did not align with theresearchtopic |
| Stoutenberg 2016[16] | Exercise training - A beneficial intervention in the treatment of alcohol use disorders? | Studies that did not align with theresearchtopic |
| Read 2001[17] | Exercise attitudes and behaviors among persons in treatment for alcohol use disorders | Studies that did not align with theresearchtopic |
| Cabé 2020[18] | Physical activity: A promising adjunctive treatment for severe alcohol use disorder | Studies that did not align with theresearchtopic |
| Georgakouli 2015[19] | Effects of acute exercise on liver function and blood redox status in heavy drinkers | Intervention is acute exercise |
| Hallgren 2021[20] | Effects of acute exercise on craving, mood and anxiety in non-treatment seeking adults with alcohol use disorder: An exploratory study | Intervention is acute exercise |
| Ussher2004[21] | Acute effect of a brief bout of exercise on alcohol urges | Intervention is acute exercise |
| Manthou 2016[22] | Role of exercise in the treatment of alcohol use disorders | Intervention is acute exercise |
| Hallgren 2021[23] | Changes in craving following acute aerobic exercise in adults with alcohol use disorder | Intervention is acute exercise |
| Torok 2023[24] | Effects of Acute Exercise on Affect in Females with Substance Use Disorder | Intervention is acute exercise |
| Murphy 1986[25] | Lifestyle Modification with Heavy Alcohol Drinkers: Effects of Aerobic Exercise and Meditation | studies with incomplete data |
| Sinyor 1981[26] | The Role of a Physical Fitness Program in theTreatment of Alcoholism | studies with incomplete data |
| Leighton 2023[27] | The Impact of an Adjunctive Group-based Exercise Programme on Alcohol Consumption and Psychological Outcomes Among Adults Attending UK Alcohol Treatment Services: A Randomised Controlled Pilot Study | studies with incomplete data |
| Brown 2016[28] | An exploratory analysis of changes in mood, anxiety and craving from pre- to postsingle sessions of exercise, over 12 weeks, among patients with alcohol dependence | Studies reusing data |
| Abrantes 2017[29] | Developing a Fitbit-Supported Lifestyle Physical Activity Intervention for Depressed Alcohol Dependent Women | Non-randomized controlled trials |
| Bichler 2017[30] | Acute effects of exercise on affective responses, cravings and heart rate variability in inpatients with alcohol use disorder e A randomized cross-over trial | Non-randomized controlled trials |
| Blodgett 2023[31] | Does moderate to vigorous physical activity mediate the association between depression and physical function in midlife: Evidence from two British birth cohort studies | Non-randomized controlled trials |
| Brown 2010[32] | A pilot study of aerobic exercise as an adjunctive treatment for drug dependence | Non-randomized controlled trials |
| capodaglio 2003[33] | A functional assessment methodology for alcohol dependent patients undergoing rehabilitative treatments | Non-randomized controlled trials |
| coiro 2007[34] | Adrenocorticotropic Hormone/Cortisol Response to Physical Exercise in Abstinent Alcoholic Patients | Non-randomized controlled trials |
| Gür 2017[35] | The Effect of Cognitive-behavioral Model-based Exercise Intervention on the Quality of Life in Alcohol-use Disorder | Non-randomized controlled trials |
| Bilberg 2019[36] | Saying yes or no to physical activity – A comparative cohort analysis of patients seeking treatment for Alcohol Use Disorder | Non-randomized controlled trials |
| Zhu 2021[37] | Effects of a Group-Based Aerobic Exercise Program on the Cognitive Functions and Emotions of Substance Use Disorder Patients: a Randomized Controlled Trial | Studies with participants not AUDs |
| Rhew 2007[38] | Effects of an exercise intervention on other health behaviors inoverweight/obese post-menopausal women | Studies with participants not AUDs |
| Colledge 2018[39] | The effects of an acute bout of exercise on neural activity in alcohol and cocaine craving: study protocol for a randomised controlled trial | Studies with participants not AUDs |

**S4 Table.** **Assessment of the risk of bias for the primary outcome.**

| Number of drinks per day | | | | | | |
| --- | --- | --- | --- | --- | --- | --- |
|  | D1 | D2 | D3 | D4 | D5 | Overall |
| Abrantes 2024 | Low | Some  concerns | Low | Low | Low | Some  concerns |
| Hallgren 2014 | Low | Low | High | Low | Low | High |
| Roessler 2017a | Low | Low | Low | Low | Low | Low |
| Roessler 2017b | Low | Low | Low | Low | Low | Low |
| Jensen 2019a | Low | Some  concerns | Some  concerns | Low | Low | Some  concerns |
| Jensen 2019b | Low | Some  concerns | Some  concerns | Low | Low | Some  concerns |
| Number of drinks per weeks | | | | | | |
|  | D1 | D2 | D3 | D4 | D5 | Overall |
| Hallgren 2014 | Low | Low | High | Low | Low | High |
| Weinstock 2014 | Low | Low | Low | Low | Low | Low |
| Weinstock 2020 | Low | Some  concerns | Low | Low | Low | Some  concerns |
| Gunillasdotter 2022a | Low | Low | Low | Low | Low | Low |
| Gunillasdotter 2022b | Low | Low | Low | Low | Low | Low |
| AUDIT | | | | | | |
|  | D1 | D2 | D3 | D4 | D5 | Overall |
| Whitworth 2019 | Low | Some  concerns | Low | Low | Low | Some  concerns |
| Reddy 2014 | Some  concerns | Some  concerns | Low | Low | Some  concerns | Some  concerns |
| Hallgren 2014 | Low | Low | High | Low | Low | High |
| Gunillasdotter 2022a | Low | Low | Low | Low | Low | Low |
| Gunillasdotter 2022b | Low | Low | Low | Low | Low | Low |

Domains:

D1: Bias arising from the randomization process.

D2: Blas due to deviations from intended intervention.

D3: Bias due to missing outcome data.

D4: Bias in measurement of the outcome.

D5: Bias in selection of the reported result.

| Maximal oxygen uptake | | | | | | |
| --- | --- | --- | --- | --- | --- | --- |
|  | D1 | D2 | D3 | D4 | D5 | Overall |
| Jensen 2019a | Low | Some  concerns | Some  concerns | Low | Low | Some  concerns |
| Jensen 2019b | Low | Some  concerns | Some  concerns | Low | Low | Some  concerns |
| Weinstock 2014 | Low | Low | Low | Low | Low | Low |
| Brown 2014 | Low | Low | Low | Low | Low | Low |
| Weinstock 2020 | Low | Some  concerns | Low | Low | Low | Some  concerns |
| Resting heart rate | | | | | | |
|  | D1 | D2 | D3 | D4 | D5 | Overall |
| Gary 1972 | Low | Some  concerns | Low | Low | Some  concerns | Some  concerns |
| McKelvy 1980 | Low | Low | Low | Low | Low | Low |
| Anxiety | | | | | | |
|  | D1 | D2 | D3 | D4 | D5 | Overall |
| Abrantes 2024 | Low | Some  concerns | Low | Low | Low | Some  concerns |
| Gur 2019 | High | Some  concerns | Low | Low | Low | High |
| Hallgren 2014 | Low | Low | High | Low | Low | High |
| Kiraz 2023 | Low | Some  concerns | Some  concerns | Low | Low | Some  concerns |
| Welford 2022a | Low | Low | Low | Low | Low | Low |
| Welford 2022b | Low | Low | Low | Low | Low | Low |
| Whitworth 2019 | Low | Some  concerns | Low | Low | Low | Some  concerns |
| Depression | | | | | | |
|  | D1 | D2 | D3 | D4 | D5 | Overall |
| Abrantes 2024 | Low | Some  concerns | Low | Low | Low | Some  concerns |
| Gür 2019 | High | Some  concerns | Low | Low | Low | High |
| Kiraz 2023 | Low | Some  concerns | Some  concerns | Low | Low | Some  concerns |
| Vedamurthachar 2006 | Some  concerns | Some  concerns | Low | Some  concerns | Low | Some  concerns |
| Weinstock 2020 | Low | Some  concerns | Low | Low | Low | Some  concerns |
| Welford 2022a | Low | Low | Low | Low | Low | Low |
| Welford 2022b | Low | Low | Low | Low | Low | Low |
| Whitworth 2019 | Low | Some  concerns | Low | Low | Low | Some  concerns |
| Perceived Stress Scale | | | | | | |
|  | D1 | D2 | D3 | D4 | D5 | Overall |
| Abrantes 2024 | Low | Some  concerns | Low | Low | Low | Some  concerns |
| Rajasekaran 2024 | Low | Low | Low | Low | Low | Low |

**S5 Table. GRADE summary of findings.**

| **Certainty Assessment** | | | | | | | | |
| --- | --- | --- | --- | --- | --- | --- | --- | --- |
| **Outcomes** | **Participants  (studies)** | **Risk of bias** | **Inconsistency** | **Indirectness** | **Imprecision** | **Publication  bias** | **Overall  certainty** | **SMD (95%CI)** |
| NDPD | 223 (4 RCTs) | serious | serious (53.5%) | not serious | serious | undetected | **⊕◯◯◯ Very low** | -0.661 (-1.033,-0.288) |
| NDPW | 233 (4 RCTs) | not serious | not serious (33.9%) | not serious | serious | undetected | **⊕⊕◯◯ Low** | -0.14 (-0.224,0.656) |
| AUDIT | 193 (4 RCTs) | serious | not serious (0%) | not serious | serious | undetected | **⊕⊕◯◯ Low** | -0.36 (0.62,-0.1) |
| VO^2^_max_ | 197 (4 RCTs) | serious | not serious (12.7%) | not serious | serious | undetected | **⊕⊕◯◯ Low** | 0.406 (0.116,0.697) |
| RHR | 68 (2 RCTs) | serious | not serious (14.5%) | not serious | serious | undetected | **⊕⊕◯◯ Low** | -0.863 (-1.438,-0.288) |
| Anxiety | 312 (6 RCTs) | serious | not serious (84.2%) | not serious | serious | undetected | **⊕◯◯◯ Very low** | -0.791 (-1.369,-0.213) |
| Depression | 422 (7 RCTs) | serious | not serious (87%) | not serious | not serious | undetected | **⊕⊕◯◯ Low** | -0.86 (-1.409,-0.31) |
| PSS | 119 (2 RCTs) | serious | not serious (93%) | not serious | serious | undetected | **⊕◯◯◯ Very low** | -2.127 (-3.91,-0.344) |

SMD: standardized mean difference; CI: confidence interval. NDPD: Number of drinks per day; NDPW: number of drinks per week; AUDIT : Alcohol Use Disorders Identification Test; VO2max:Maximal oxygen uptake; RHA: Resting heart rate; PSS: Perceived Stress Scale.

GRADE Working Group grades of evidence:

High quality: Further research is very unlikely to change our confidence in the estimate of effect.

Moderate quality: Further research is likely to have an important impact on our confidence in the estimate of effect and may change the estimate.

Low quality: Further research is very likely to have an important impact on our confidence in the estimate of effect and is likely to change the estimate.

Very low quality: We are very uncertain about the estimate.

**References**

1. Brown RA, Abrantes AM, Read JP, et al. Aerobic exercise for alcohol recovery: rationale, program description, and preliminary findings. Behav Modif. 2009;33(2): 220–249. https://doi:10.1177/0145445508329112PMID:19091721

2. Giesen ES, Zimmer P, Bloch W. Effects of an Exercise Program on Physical Activity Level and Quality of Life in Patients with Severe Alcohol Dependence. Alcoholism Treatment Quarterly. 2016;34(1): 63–78. https://doi:10.1080/07347324.2016.1113109

3. Hallgren M, Andersson V, Ekblom Ö, et al. Physical activity as treatment for alcohol use disorders (FitForChange): study protocol for a randomized controlled trial. Trials. 2018;19(1): 106. https://doi:10.1186/s13063-017-2435-0PMID:29444712

4. Hovhannisyan K, Rasmussen M, Adami J, et al. Evaluation of Very Integrated Program: Health Promotion for Patients With Alcohol and Drug Addiction-A Randomized Trial. Alcohol Clin Exp Res. 2020;44(7): 1456–1467. https://doi:10.1111/acer.14364PMID:32424821

5. Piorkowski M, Axtell LA. Effect of circuit exercise training on physical fitness of alcoholic patients. Physical therapy. 1976;56(4): 403–406.

6. Roessler KK, Mau M, Ekstrøm CT. Interpersonal problems of alcohol use disorder patients undergoing a physical exercise intervention – a randomised controlled trial. Nordic Psychology. 2018;70(3): 245–255. https://doi:10.1080/19012276.2017.1418414

7. Sari S, Bilberg R, Søgaard Nielsen A, et al. The effect of exercise as adjunctive treatment on quality of life for individuals with alcohol use disorders: a randomized controlled trial. BMC Public Health. 2019;19(1): 727. https://doi:10.1186/s12889-019-7083-8PMID:31185955

8. Weinstock J, Petry NM, Pescatello LS, et al. Sedentary college student drinkers can start exercising and reduce drinking after intervention. Psychol Addict Behav. 2016;30(8): 791–801. https://doi:10.1037/adb0000207PMID:27669095

9. Houghton D, Hallsworth K, Thoma C, et al. Effects of Exercise on Liver Fat and Metabolism in Alcohol Drinkers. Clin Gastroenterol Hepatol. 2017;15(10): 1596-1603.e3. https://doi:10.1016/j.cgh.2017.05.001PMID:28501537

10. Williams PT. The relationships of vigorous exercise, alcohol, and adiposity to low and high high-density lipoprotein-cholesterol levels. Metabolism. 2004;53(6): 700–709. https://doi:10.1016/j.metabol.2004.01.004PMID:15164315

11. El-Sayed MS, Ali N, El-Sayed Ali Z. Interaction between alcohol and exercise: physiological and haematological implications. Sports Med. 2005;35(3): 257–269. https://doi:10.2165/00007256-200535030-00005PMID:15730339

12. Yokoyama M, Yokoyama T, Funazu K, et al. Associations between headache and stress, alcohol drinking, exercise, sleep, and comorbid health conditions in a Japanese population. J Headache Pain. 2009;10(3): 177–185. https://doi:10.1007/s10194-009-0113-7PMID:19326184

13. Müller A, Loeber S, Söchtig J, et al. Risk for exercise dependence, eating disorder pathology, alcohol use disorder and addictive behaviors among clients of fitness centers. J Behav Addict. 2015;4(4): 273–280. https://doi:10.1556/2006.4.2015.044PMID:26690622

14. Fadus MC, Squeglia LM, Valadez EA, et al. Adolescent Substance Use Disorder Treatment: an Update on Evidence-Based Strategies. Curr Psychiatry Rep. 2019;21(10): 96. https://doi:10.1007/s11920-019-1086-0PMID:31522280

15. Suter PM, Schutz Y. The effect of exercise, alcohol or both combined on health and physical performance. Int J Obes (Lond). 2008;32 Suppl 6: S48-52. https://doi:10.1038/ijo.2008.206PMID:19079280

16. Stoutenberg M, Rethorst CD, Lawson O, et al. Exercise training - A beneficial intervention in the treatment of alcohol use disorders? Drug Alcohol Depend. 2016;160: 2–11. https://doi:10.1016/j.drugalcdep.2015.11.019PMID:26652900

17. Read JP, Brown RA, Marcus BH, et al. Exercise attitudes and behaviors among persons in treatment for alcohol use disorders. J Subst Abuse Treat. 2001;21(4): 199–206. https://doi:10.1016/s0740-5472(01)00203-3PMID:11777669

18. Cabé N, Lanièpce A, Pitel AL. Physical activity: A promising adjunctive treatment for severe alcohol use disorder. Addict Behav. 2021;113: 106667. https://doi:10.1016/j.addbeh.2020.106667PMID:33074123

19. Georgakouli K, Manthou E, Fatouros IG, et al. Effects of acute exercise on liver function and blood redox status in heavy drinkers. Exp Ther Med. 2015;10(6): 2015–2022. https://doi:10.3892/etm.2015.2792PMID:26668589

20. Hallgren M, Vancampfort D, Hoang MT, et al. Effects of acute exercise on craving, mood and anxiety in non-treatment seeking adults with alcohol use disorder: An exploratory study. Drug Alcohol Depend. 2021;220: 108506. https://doi:10.1016/j.drugalcdep.2021.108506PMID:33461151

21. Ussher M, Sampuran AK, Doshi R, et al. Acute effect of a brief bout of exercise on alcohol urges. Addiction. 2004;99(12): 1542–1547. https://doi:10.1111/j.1360-0443.2004.00919.xPMID:15585045

22. Manthou E, Georgakouli K, Fatouros IG, et al. Role of exercise in the treatment of alcohol use disorders. Biomed Rep. 2016;4(5): 535–545. https://doi:10.3892/br.2016.626PMID:27123244

23. Hallgren M, Herring MP, Vancampfort D, et al. Changes in craving following acute aerobic exercise in adults with alcohol use disorder. J Psychiatr Res. 2021;142: 243–249. https://doi:10.1016/j.jpsychires.2021.08.007PMID:34391078

24. Torok VA, Brewer CB, Lake HN. Effects of Acute Exercise on Affect in Females with Substance Use Disorder. Int J Exerc Sci. 2023;16(5): 95–108.

25. Murphy TJ, Pagano RR, Marlatt GA. Lifestyle modification with heavy alcohol drinkers: effects of aerobic exercise and meditation. Addict Behav. 1986;11(2): 175–186. https://doi:10.1016/0306-4603(86)90043-2PMID:3526824

26. Sinyor D, Brown T, Rostant L, et al. The role of a physical fitness program in the treatment of alcoholism. J Stud Alcohol. 1982;43(3): 380–386. https://doi:10.15288/jsa.1982.43.380

27. Leighton D, Foster G, Wills W, et al. P9 The impact of an adjunctive group-based exercise programme on alcohol consumption and psychological outcomes among adults attending UK alcohol treatment services: a randomised controlled pilot study. BMJ Publishing Group; 2023.

28. Brown RA, Prince MA, Minami H, et al. An exploratory analysis of changes in mood, anxiety and craving from pre- to post-single sessions of exercise, over 12 weeks, among patients with alcohol dependence. Ment Health Phys Act. 2016;11: 1–6. https://doi:10.1016/j.mhpa.2016.04.002PMID:29606975

29. Abrantes AM, Blevins CE, Battle CL, et al. Developing a Fitbit-supported lifestyle physical activity intervention for depressed alcohol dependent women. J Subst Abuse Treat. 2017;80: 88–97. https://doi:10.1016/j.jsat.2017.07.006PMID:28755778

30. Bichler C, Niedermeier M, Fruehauf A, et al. Acute effects of exercise on affective responses, cravings and heart rate variability in inpatients with alcohol use disorder–a randomized cross-over trial. Mental Health and Physical Activity. 2017;13: 68–76.

31. Blodgett JM, Norris T, Pinto Pereira SM, et al. Does moderate to vigorous physical activity mediate the association between depression and physical function in midlife: Evidence from two British birth cohort studies. J Affect Disord. 2023;326: 206–215. https://doi:10.1016/j.jad.2022.12.084PMID:36584709

32. Brown RA, Abrantes AM, Read JP, et al. A Pilot Study of Aerobic Exercise as an Adjunctive Treatment for Drug Dependence. Ment Health Phys Act. 2010;3(1): 27–34. https://doi:10.1016/j.mhpa.2010.03.001PMID:20582151

33. Capodaglio EM, Vittadini G, Bossi D, et al. A functional assessment methodology for alcohol dependent patients undergoing rehabilitative treatments. Disabil Rehabil. 2003;25(21): 1224–1230. https://doi:10.1080/09638280310001608573PMID:14578062

34. Coiro V, Casti A, Jotti GS, et al. Adrenocorticotropic hormone/cortisol response to physical exercise in abstinent alcoholic patients. Alcohol Clin Exp Res. 2007;31(5): 901–906. https://doi:10.1111/j.1530-0277.2007.00376.xPMID:17386066

35. Gür F, Can Gür G, Okanlı A. The Effect of the Cognitive-behavioral Model-based Psychoeducation and Exercise Intervention on Quality of Life in Alcohol Use Disorder. Arch Psychiatr Nurs. 2017;31(6): 541–548. https://doi:10.1016/j.apnu.2017.07.005PMID:29179818

36. Bilberg R, Roessler KK, Nielsen AS. Saying yes or no to physical activity - A comparative cohort analysis of patients seeking treatment for Alcohol Use Disorder. Addict Behav Rep. 2019;9: 100180. https://doi:10.1016/j.abrep.2019.100180PMID:31193872

37. Zhu T, Tao W, Peng B, et al. Effects of a Group-Based Aerobic Exercise Program on the Cognitive Functions and Emotions of Substance Use Disorder Patients: a Randomized Controlled Trial. Int J Ment Health Addiction. 2022;20(4): 2349–2365. https://doi:10.1007/s11469-021-00518-x

38. Rhew I, Yasui Y, Sorensen B, et al. Effects of an exercise intervention on other health behaviors in overweight/obese post-menopausal women. Contemp Clin Trials. 2007;28(4): 472–481. https://doi:10.1016/j.cct.2007.01.002PMID:17287149

39. Colledge F, Ludyga S, Mücke M, et al. The effects of an acute bout of exercise on neural activity in alcohol and cocaine craving: study protocol for a randomised controlled trial. Trials. 2018;19(1): 713. https://doi:10.1186/s13063-018-3062-0PMID:30594237
